# *Arabidopsis* NSE4 Proteins Act in Somatic Nuclei and Meiosis to Ensure Plant Viability and Fertility

**DOI:** 10.3389/fpls.2019.00774

**Published:** 2019-06-20

**Authors:** Mateusz Zelkowski, Katarzyna Zelkowska, Udo Conrad, Susann Hesse, Inna Lermontova, Marek Marzec, Armin Meister, Andreas Houben, Veit Schubert

**Affiliations:** ^1^Leibniz Institute of Plant Genetics and Crop Plant Research, Gatersleben, Germany; ^2^Plant Cytogenomics Research Group, Central European Institute of Technology, Masaryk University, Brno, Czechia; ^3^Department of Genetics, Faculty of Biology and Environmental Protection, University of Silesia, Katowice, Poland

**Keywords:** *Arabidopsis thaliana*, meiosis, mitosis, NSE4 δ-kleisin, nucleus, phylogeny, SMC5/6 complex, super-resolution microscopy

## Abstract

The SMC 5/6 complex together with cohesin and condensin is a member of the structural maintenance of chromosome (SMC) protein family. In non-plant organisms SMC5/6 is engaged in DNA repair, meiotic synapsis, genome organization and stability. In plants, the function of SMC5/6 is still enigmatic. Therefore, we analyzed the crucial δ-kleisin component NSE4 of the SMC5/6 complex in the model plant *Arabidopsis thaliana*. Two functional conserved *Nse4* paralogs (*Nse4A* and *Nse4B*) are present in *A. thaliana*, which may have evolved via gene subfunctionalization. Due to its high expression level, *Nse4A* seems to be the more essential gene, whereas *Nse4B* appears to be involved mainly in seed development. The morphological characterization of *A. thaliana* T-DNA mutants suggests that the NSE4 proteins are essential for plant growth and fertility. Detailed investigations in wild-type and the mutants based on live cell imaging of transgenic GFP lines, fluorescence *in situ* hybridization (FISH), immunolabeling and super-resolution microscopy suggest that NSE4A acts in several processes during plant development, such as mitosis, meiosis and chromatin organization of differentiated nuclei, and that NSE4A operates in a cell cycle-dependent manner. Differential response of NSE4A and NSE4B mutants after induced DNA double strand breaks (DSBs) suggests their involvement in DNA repair processes.

## Introduction

The evolutionarily conserved structural maintenance of chromosome (SMC) protein complexes are ubiquitous across different organisms from bacteria to humans, and act in basic biological processes such as sister chromatid cohesion, chromosome condensation, transcription, replication, DNA repair and recombination. The SMC proteins realize these many different functions via ATP-stimulated DNA-bridging to perform intra- and intermolecular linking. Together with non-SMC proteins, including kleisin subunits, SMC proteins form ring-shaped multi-protein complexes, such as cohesins, condensins and SMC5/6 complexes (Nasmyth and Haering, 2005; Hirano, 2006; Jeppsson et al., 2014b;Haering and Gruber, 2016a,b).

It has been proposed that a bacterial or archaea SMC is the forerunner of all eukaryotic SMC complexes. Due to its interactions with the conserved kite (kleisin-interacting tandem winged-helix elements) proteins the SMC5/6 complex is regarded to represent the closest eukaryotic relative to the common SMC ancestor compared to cohesin and condensin (Palecek and Gruber, 2015).

SMC5/6 complexes are formed through the interaction of the hinge domains of the SMC5 and SMC6 proteins resulting in a heterodimer connected by the δ-kleisin NSE4 (NON-SMC ELEMENT 4) at the head domains of SMC5 and SMC6. In human and yeasts six (NSE1–6) different non-SMC elements were identified (Fousteri and Lehmann, 2000; Hazbun et al., 2003; Palecek et al., 2006; Taylor et al., 2008; Räschle et al., 2015).

Originally, the SMC5/6 complex has mainly been investigated for its function in DNA repair (Lehmann, 2005) by regulating homologous recombination at DNA breaks, stalled replication forks and rDNA (Torres-Rosell et al., 2005, 2007a,b; De Piccoli et al., 2006; Lindroos et al., 2006; Irmisch et al., 2009). In yeast, together with cohesin, SMC5/6 is involved in DSB repair to manage proper sister chromatid segregation (Uhlmann and Nasmyth, 1998; Sjögren and Nasmyth, 2001; Ünal et al., 2004; Torres-Rosell et al., 2005; De Piccoli et al., 2006). Similarly, in human cells, SMC5/6 is also involved in the recruitment of cohesin to DSB sites (Potts et al., 2006).

Furthermore, SMC5/6 facilitates the resolution of sister chromatid intertwinings and replication-induced DNA supercoiling to allow correct chromosome segregation (Bermúdez-López et al., 2010; Kegel et al., 2011; Gallego-Paez et al., 2014; Jeppsson et al., 2014a). The complex is required for telomere maintenance (Zhao and Blobel, 2005; Potts and Yu, 2007), and it has been found that SMC5/6 regulates chromosome stability and dynamics via ATP-regulated intermolecular DNA linking (Kanno et al., 2015).

The involvement of SMC5/6 components in DNA repair pathways and in activities known from cohesin, condensin indicates that SMC5/6 has a key role in maintaining chromosome stability (De Piccoli et al., 2009). The participation of SMC5/6 in cohesin- and condensin-like functions indicates that these functions seem to be realized via the DNA-bridging activity of SMC5/6, and/or through direct or indirect control of the other two complexes (Jeppsson et al., 2014b).

In addition to functions of SMC5/6 in somatic tissues, different essential roles during meiosis were proven in model organisms as yeasts, worm, mouse and human. The data indicate the involvement of SMC5/6 components in such meiotic processes as response to DSBs, meiotic recombination, heterochromatin maintenance, centromere cohesion, homologous chromosome synapsis and meiotic sex chromosome inactivation (Verver et al., 2016).

**FIGURE 1:**
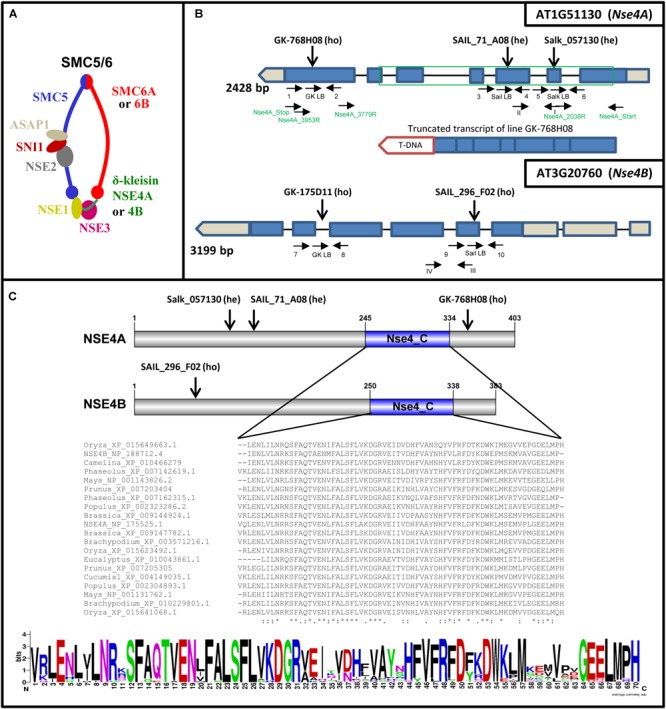
*A. thaliana* SMC5/6 complexes and their δ-kleisin subunits NSA4A and NSE4B. **(A)** Subunit composition of SMC5/6 complexes based on a model according to Nasmyth and Haering (2005) and Schubert (2009). The SMC5/6 complexes presumably have one SMC5 subunit, two alternative SMC6 subunits, the NSE1, NSE2, NSE3, NSE5-like (SNI1), NSE6-like (ASAP1) subunits, and in addition, the two different δ-kleisins NSA4A and NSE4B. The sub-complexes NSE2-SNI1-ASAP1, NSE1-NSE3-NSE4, and SNI1-ASAP1 may act as specialized functional modules (Sergeant et al., 2005; Palecek et al., 2006; Duan et al., 2009). **(B)** Schematic view of the *Nse4A* and *Nse4B* gene structures (mips.helmholtz-muenchen.de, Version 10; ncbi.nlm.nih.gov; pfam.sanger.ac.uk) and the expressed truncated transcript of the T-DNA line GK-768H08. Exons are shown as blue boxes. UTRs are visible in gray. The green frame indicates the region used for recombinant protein expression and the production of antibodies. The T-DNA insertions (*A. thaliana* SALK, SAIL, and GK lines) and gene-specific primers used for genotyping are indicated by arrows. Arabic numbers indicate gene-specific primers used for genotyping. Roman numbers denote primers applied for RT and real-time PCR. The primers used to confirm the truncated transcript of line GK-768H08 (T-DNA insertion visualized as red box) are indicated in green (see also Supplementary Figure S9). **(C)** Top: schematic view of the NSE4A and NSE4B protein structures. The conserved NSE4_C motif and the T-DNA insertion positions are indicated. Middle: Alignment of the NSE4_C motifs present in putative NSE4 orthologs of higher plants. The alignment was performed by the Clustal Omega 2.1 software. ^∗^, Identical amino acids; :, similar amino acids; –, missing amino acids. Bottom: the same alignment as above presented in the sequence logo format (WebLogo; http://weblogo.berkeley.edu/logo.cgi) to compare similarities and differences in all selected sequences of the NSE4_C motif more easily.

Similar as in other organisms, SMC complexes are also present in plants to perform different essential functions together with interacting factors (Schubert, 2009; Diaz and Pecinka, 2018). Due to the presence of two alternative SMC6 (SMC6A and SMC6B) and δ-kleisin NSE4 (NSE4A and NSE4B) subunits in *Arabidopsis thaliana*, different SMC5/6 complexes may be formed (Figure 1A). While NSE1-4 are highly conserved in eukaryotes, NSE5 and NSE6 are not conserved at the DNA sequence level. Nevertheless, based on protein complex purification and interaction data the proteins ASAP1 and SNI1 were suggested to be the functional *A. thaliana* counterparts of NSE5 and NSE6 found in other multicellular organisms (Yan et al., 2013).

SMC5, SMC6A, and SMC6B are required together with SYN1 (the α-kleisin of *A. thaliana* cohesin) to align sister chromatids after DNA breakage, apparently to facilitate repair via homologous recombination in somatic cells (Mengiste et al., 1999; Hanin et al., 2000; Watanabe et al., 2009). The *A. thaliana* SUMO E3 ligase AtMMS21 (a homolog of NSE2) regulates cell proliferation in roots via cell-cycle regulation and cytokinin signaling (Huang et al., 2009), and is involved in root stem cell niche maintenance and DNA damage responses (Xu et al., 2013). NSE1 and NSE3 of *A. thaliana* have a role in DNA damage repair and are required for early embryo and seedling development (Li et al., 2017). Transcripts of *Nse4A* but not of *Nse4B* were detected in seedlings, rosette leaves, and immature flower buds, suggesting that *Nse4A* is a functional gene in *A. thaliana* cells (Watanabe et al., 2009).

However, the biological function of the two *A. thaliana* NSE4 homologs has not yet been determined in detail. Here we show that both genes are essential for plant growth and fertility. Via applying live cell imaging, FISH, immunolabeling and super-resolution microscopy, we found that especially NSE4A proteins act in transcriptionally active somatic interphase chromatin and that they are essential for proper mitosis and meiosis.

## Materials and Methods

### Plant Material and Genotyping

The *A. thaliana* (L.) Heynh. SALK and SAIL T-DNA insertion lines in ecotype Columbia (Col-0) were obtained from the Salk Institute, Genomic Analysis Laboratory^1^ (Alonso et al., 2003) and from the Syngenta collection of T-DNA insertion mutants (Sessions et al., 2002), respectively. The GABI T-DNA mutants (GK in Col-0) were generated in the context of the GABI-Kat program (MPI for Plant Breeding Research, Cologne, Germany^2^; Rosso et al., 2003). All lines were provided by the Nottingham Arabidopsis Stock Centre^3^.

Seeds were germinated in soil followed by cultivation under short day conditions (8 h light/16 h dark) at 18°C. After 1 month the plants were transferred to long day conditions (16 h light, 22°C/8 h dark, 21°C). Genomic DNA was isolated from rosette leaves and used for PCR-based genotyping to identify heterozygous and homozygous T-DNA insertion mutants. The PCR primers used for genotyping are listed in Supplementary Table S1, and their positions are shown in Figure 1B. The following PCR program was used: initial denaturation for 5 min at 95°C, then 40 cycles with 15 s denaturation at 95°C, 30 s annealing at 55°C, and 60 s final elongation at 72°C.

Polymerase chain reaction using the gene-specific primer sets yielded DNA fragments of ∼1 kb representing the wild-type alleles. The PCR fragments specific for the disrupted allele yielded PCR products of ∼0.5 kb. The positions of T-DNA insertion were confirmed by sequencing the PCR-amplified T-DNA junction fragments (Supplementary Table S2).

To obtain double T-DNA insertion mutants cross-fertilization was performed.

*Brassica rapa* L. plants were grown under long day conditions (16 h light, 22°C/8 h dark, 18°C) to obtain meiocytes for immunolocalization of NSE4A via specific antibodies.

### *In silico* Analysis of Gene and Protein Structures and the Phylogenetic Tree Construction

Gene structures of NSE4A and NSE4B were predicted at mips.helmholtz-muenchen.de (Version 10 ^4, 5^). The conserved functional domains of known putative NSE4 orthologs of higher plants (full-length sequences are available at www.ncbi.nlm.nih.gov/) were identified using the Conserved Domain Database^6^. The same sequences were used to generate a phylogenetic tree by Bayesian phylogenetic inference in MrBayes 3.2.6 ^7^. All alignments were performed by the Clustal Omega 2.1 software^8^.

### Gene Expression Analysis

Total RNA was isolated from seedlings, three and 6 weeks old leaves, flower buds, and root tissues using the Trizol (Thermo Fisher Scientific) method according to manufacturer’s instructions. Then, the samples were DNase-treated applying the TURBO DNA-free^TM^ Kit (Thermo Fisher Scientific). Reverse transcription (RT) was performed using the random hexamer RevertAid Reverse Transcriptase Kit (Thermo Fisher Scientific). After 5 min initial denaturation at 95°C, followed by 60 min cDNA synthesis at 42°C, the reaction was terminated at 70°C for 5 min.

Quantitative real-time PCR with SYBR Green was performed using a QuantStudio 5 flex machine and the QuantStudio^TM^ Real-Time PCR Software (v1.1). One microliter of cDNA was applied for each reaction with three replicates and three independent biological repetitions for each tissue or developmental stage. The following PCR program was used: initial denaturation for 5 min at 95°C, then 40 cycles with 15 s denaturation at 95°C, 30 s annealing at 60°C, and 20 s final elongation at 72°C. *PP2A* (AT1G13320) and *RHIP1* (AT4G26410) served as standards (Czechowski et al., 2005). Calculations were based on the delta CT values of the reference genes (Livak and Schmittgen, 2001). The quantitative real-time RT-PCR primers used to amplify transcripts are shown in Figure 1B and Supplementary Table S3.

### Cloning and Transformation

PCR-based amplification of cDNA (for 35S::*Nse4A*::EYFP) and genomic DNA (for promoterNse4A::gNse4A::GFP) as templates were performed using the KOD Xtreme^TM^ Hot Start DNA Polymerase (Merck). The PCR products were cloned into the pJET 1.2 vector using the CloneJET PCR Cloning Kit (Thermo Fisher Scientific). Sequence-confirmed inserts were cloned into the Gateway^®^ pENTR^TM^ 1A Dual Selection Vector (Thermo Fisher Scientific). Next, the inserts were re-cloned into the pGWG (complementation vector without promoter and tag), pGWB642 (35S promoter with EYFP tag on N-term) and pGWB604 (no promoter, GFP-tag on C-terminus) vectors (Neyagawa vectors, doi.org/10.1271/bbb.100184; Nakamura et al., 2010) using the BP Clonase II kit (Gateway^®^ Technology, Thermo Fisher Scientific). The binary vectors were transferred into *Agrobacterium tumefaciens*, and then used to transform *A. thaliana* Col-0 wild-type plants via the floral dip method (Clough and Bent, 1998). Seeds from these plants were propagated on PPT medium (16 μg/ml). Positively selected seedlings were transferred into soil and genotyped for the presence of the construct. Homozygous F2 plants were used in further studies. Primers used for the cloning are listed in Supplementary Table S4.

### Recombinant Protein and Antibody Production

For antibody production the partial NSE4A peptide (from 49 to 289 aa) (Supplementary Figure S1) was expressed in the *E. coli* BL21 pLysS strain using the pET23a (Novagen) vector. Primers used for the recombinant protein production are listed in Supplementary Table S4. The recombinant proteins containing 6xHis-tags were purified using Dynabeads His-Tag (Thermo Fisher Scientific) according to manufacturer’s instructions. Five hundred microliters cleared extract was mixed with 500 μl binding buffer (50 mM NaP, pH 8.0, 300 mM NaCl, 0.01% Tween-20), and 50 μl washed Dynabeads were added. After 10 min incubation on a roller, the beads were washed 7 × with binding buffer, and 7 × with binding buffer, 5 mM imidazole. The elution was done with binding buffer, 150 mM imidazole, and the protein concentration (90 ng/μl) was determined using a Bradford kit (Bio-Rad Laboratories GmbH, Munich) (Bradford, 1976).

The separation on SDS gels and the protein size determination by Western analysis was done as described (Conrad et al., 1997; Supplementary Figure S2A).

Two rabbits were immunized with 1 mg NSE4A protein and complete Freund’s adjuvants. Four and five weeks later, booster immunizations were performed with 0.5 mg NSE4A protein and incomplete Freund’s adjuvants, respectively. Ten days later blood was taken, serum isolated, precipitated in 40% saturated ammonium sulfate, dialysed against 1 × PBS and affinity purified.

The specific binding behavior of the rabbit anti-NSE4 antibodies was investigated by competitive ELISA according to Conrad et al. (2011). The wells were coated with 46 ng/100 μl recombinant affinity-purified NSE4A in 1 × PBS and incubated overnight at room temperature. After blocking with 3% w/v BSA in 1 × PBS-0.05% w/v Tween 20 (1 × PBS-T) for 2 h, the known amounts of affinity-purified anti-NSE4A antibodies were mixed with various concentrations of NSE4A in 1% w/v BSA in 1 × PBS-T, incubated for 30 min in a master plate, added to the antigen-coated wells and incubated for 1 h at 25°C. Antibodies bound to the plate were visualized with anti-rabbit-IgG alkaline phosphatase diluted in 1 × PBS-T/1% BSA. The enzymatic substrate was pNP phosphate, and the absorbance (405 nm) was measured after 30 min incubation at 37°C (Supplementary Figure S2B).

To further prove the NSE4A antibody specificity in immuno-histological experiments antigen competition experiments were performed. NSE4A was added to the antibodies at a concentration of 800 nM, and applied to flow-sorted 8C *A. thaliana* interphase nuclei. The signal reduction compared to the control nuclei without addition of antigen clearly confirmed the specificity (Supplementary Figure S2C).

### Complementation Assay

To confirm that the phenotypes of the of *Nse4A* mutant GK-768H08 are indeed caused by this mutation we complemented the mutant by the genomic wild-type *Nse4A* gene. The genomic intron-exon containing *Nse4A* gene with a 1.7 kbp-long upstream promoter region was amplified by PCR using the KOD Xtreme^TM^ Hot Start DNA Polymerase (Merck), and then sequenced. Next, it was cloned into the pBWG vector (Nakamura et al., 2010), and transformed into *A. tumefaciens*. Plant transformation was performed by the bacteria-mediated vector transfer via the floral dipping method (Clough and Bent, 1998), and afterward propagated under long-day conditions. The harvested seeds were grown on selective PPT medium (16 μg/ml), and positively selected seedlings were transferred to soil and genotyped for the presence of the construct. Homozygous F2 plants were used in further studies.

### Fertility Evaluation and Alexander Staining

**FIGURE 2:**
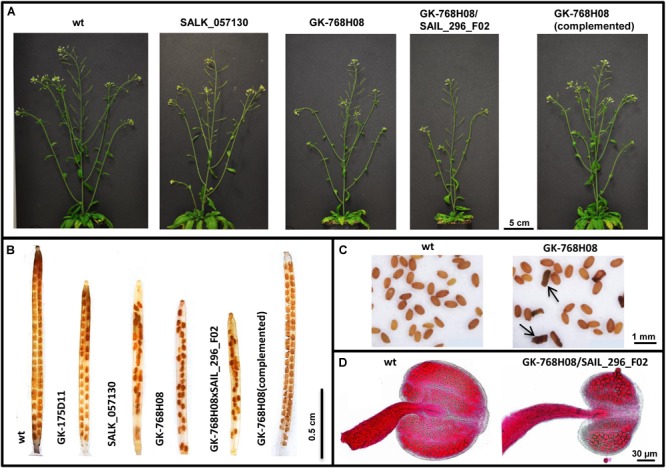
Impaired growth and fertility of *nse4* mutants compared to wild-type (wt). **(A)** Reduced plant size of the mutants GK-768H08 and the double mutant GK-768H08/SAIL_296_F02. Mutant SALK_057130 and the complemented GK-768H08 mutant show a wild-type habit. **(B)** Reduced seed set per silique in the nse4A and nse4B mutants. **(C)** Shriveled seeds (arrows) of the GK-768H08 mutant. **(D)** Reduced pollen grain number and aborted pollen grains in an anther of the double mutant GK-768H08/SAIL_296_F02.

Mature dry siliques were collected to evaluate silique length and seed setting. The seeds were classified into normal and shriveled (Figure 2). For clearing, fully developed green siliques were treated in an ethanol:acetic acid (9:1) solution overnight at room temperature, then washed in 70 and 90% ethanol for 5 min each, followed by storage in a chloral hydrate:glycerol:water (8:1:3) solution at 4°C.

To evaluate anther shape and pollen viability, Alexander staining (Alexander, 1969) was performed. Undamaged anthers were used for total pollen (per anther) counting. Afterward, the anthers were squashed and the released pollen grains were evaluated into two classes: normal (viable, pink round grains), and aborted (gray/green abnormal shape).

Images from siliques, seeds and anthers were acquired using a Nikon SMZ1500 binocular and the NIS-Elements AR 3.0 software.

### Bleomycin Treatment

To induce DNA DSBs via bleomycin application *A. thaliana* wild-type and NSE4A mutant seeds were sterilized 10 min in 70% ethanol, then 15 min in 4% Na-hypochlorite + 1 drop Tween-20, followed by washing 3 × 5 min in sterile water. The seeds were germinated on wet filter paper for 5 days, and then placed in liquid germination medium (Murashige and Skoog, Duchefa, prod. no. M0231.0025; 10 g/l sucrose, 500 mg/l MES, pH 5.7) without and with bleomycin (bleomycin sulfate from *Streptomyces verticillus*, Sigma, cat. no. 15361) of increasing concentration. Accordingly, in a second experiment the sterilized seeds were grown on agar plates (germination medium + 2% agar-agar; Roth, cat. no. 2266.2) without and with bleomycin. Both experiments were repeated twice and contained two repetitions.

### Immunostaining and FISH

Flower bud fixation, chromosome slide preparation, and FISH followed by chiasma counting were performed according to Sánchez-Morán et al. (2001). To identify individual chromosomes, 5S and 45S rDNA FISH was performed.

Fluorescence *in situ* hybridization with telomere- and centromere-specific probes was applied to identify chromosomes at metaphase I. The 180-bp centromeric repeat probe (pAL) (Martinez-Zapater et al., 1986) was generated by PCR as previously described (Kawabe and Nasuda, 2005). The telomere-specific probe was generated by PCR in the absence of template DNA using the primers (TAAACCC)_7_ and (GGGTTTA)_7_ (Ijdo et al., 1991).

Immunostaining of *A. thaliana* and *B. rapa* PMCs followed the protocol of Armstrong and Osman (2013). The following primary antibodies were applied: rabbit anti-NSE4A (1:250) and rat anti-ZYP1 (1:1000; kindly provided by Chris Franklin). ZYP1 is the *A. thaliana* transverse filament protein of the synaptonemal complex (Higgins et al., 2005). The primary antibodies were detected by donkey anti-rabbit-Alexa488 (Dianova, no. 711545152) and goat anti-rat-DyLight594 (Abcam, no. ab98383), respectively, as secondary antibodies.

8C leaf interphase nuclei were flow sorted according to Weisshart et al. (2016), and also immuno-labeled against NSE4A as described above.

### Microscopy

To image fixed and live cell preparations an Olympus BX61 microscope (Olympus) and a confocal laser scanning microscope LSM 780 (Carl Zeiss GmbH), respectively, were used.

To analyze the ultrastructure of immunosignals and chromatin beyond the classical Abbe/Raleigh limit at a lateral resolution of ∼120 nm (super-resolution, achieved with a 488 nm laser) spatial structured illumination microscopy (3D-SIM) was applied using a 63 × 1.4NA Oil Plan-Apochromat objective of an Elyra PS.1 microscope system and the software ZEN (Carl Zeiss GmbH). Images were captured separately for each fluorochrome using the 561, 488, and 405 nm laser lines for excitation and appropriate emission filters (Weisshart et al., 2016).

## Results

### Two Conserved *Nse4* Genes Are Present and Expressed in *A. thaliana*

According to previous SMC5/6 subunit prediction studies (Schubert, 2009) *A. thaliana* encodes two *Nse4* homologs: *Nse4A* (AT1G51130) and *Nse4B* (AT3G20760) (Figure 1A,B). Both NSE4 proteins show similar lengths (NSE4A: 403 aa; NSE4B: 383 aa), and a high amino acid sequence identity (67.7%) (Supplementary Figure S1). Both *A. thaliana* NSE4 proteins show similar lengths as those of budding yeast (402 aa), mouse (381 aa for NSE4A; 375 aa for NSE4B), and human NSE4A (385 aa), but are longer than the fission yeast NSE4 (300 aa) and the human NSE4B (333 aa) proteins (NSE4A^9^; NSE4B^10^).

NSE4A shows a relatively high amino acid similarity compared to both *B. rapa* putative NSE4 proteins (Supplementary Figure S3), and other plant species (Supplementary Figure S4A). Non-plant organisms such as fission yeast, *Entamoeba, Dictyostelium*, mouse and human display a lower similarity (Supplementary Table S5).

The phylogenetic analysis of the full-length protein sequences of eudicot and monocot species suggests also a relatively high conservation of both *A. thaliana Nse4* genes (Supplementary Figure S4B).

According to Uniprot databases^11^, both *A. thaliana* NSE4 proteins possess conserved C-terminal domains typical for other plant NSE4 proteins (Figure 1C and Supplementary Figures S1, S3). The C-terminal domain binds to SMC5 in the similar way as the other kleisin molecules interact with their kappa-SMC partners (Palecek et al., 2006; Hassler et al., 2018). This interaction is crucial for the function of SMC5/6. The NSE4 N-terminal domain is also conserved and binds to SMC6 (Palecek et al., 2006). In NSE4 of fungi and vertebrates, a NSE3/MAGE binding domain was identified next to the N-terminal kleisin motif (Guerineau et al., 2012). Based on the Motif Scan analysis^12^ the SMC6-binding domain can also be predicted in the NSE4 proteins of *A. thaliana* (Supplementary Figure S1). However, to define this identified region as the SMC6-binding motif clearly, protein–protein interaction, domain dissection and mutagenesis experiments have to be performed. Additionally, putative degradation regions and SUMOlisation sites were identified using Eukaryotic Linear Motif^13^ resources (Supplementary Figure S1), suggesting that the cellular amount of NSE4 proteins during the cell cycle might be regulated via their proteolytic degradation.

*In silico* analysis shows a similar expression behavior (with peaks at the young rosette and flowering stages) during plant development of the *Nse4A* gene and other SMC5/6 subunit candidate genes, supporting a synchronized activity (Supplementary Figure S5). However, it is not clear whether they act separately or as multi-subunit complexes in various subunit combinations. *In silico* analysis indicated also a high co-expression of *Nse4A*, among others, with meiosis- and chromatin-related genes (Supplementary Table S6).

The *in silico* analysis of the relative expression level of *Nse4A* and *Nse4B* in ten anatomical parts of *A. thaliana* seedlings displayed that the expression of *Nse4B* is limited to generative tissues and seeds. A relatively high expression is evident only in seeds (embryo and especially endosperm) (Supplementary Figure S6).

By quantitative real-time PCR we found that *Nse4A* is highly expressed in flower buds and roots, but transcripts are also present in seedlings, young and old leaves (Supplementary Figure S7). In agreement with previous studies (Watanabe et al., 2009), the expression of *Nse4B* in these tissues is not detectable. Obviously, most *Nse4B* transcripts are present in already well developed seeds, as also indicated by *in silico* analysis (Supplementary Figure S6).

To figure out whether the NSE4 proteins interact with the other components of the SMC5/6 complex (Figure 1) a protein-protein interactions analysis was performed *in silico* using the STRING program^14^. Interestingly, all SMC5/6 subunits accessible via the STRING program were identified as interacting partners of the NSE proteins at a very high score >0.95, suggesting that both NSE4A and NSE4B act also within the SMC5/6 complex. In addition, cohesin and condensin subunits were detected as parts of the same protein-protein interaction network at the high score of >0.70 (Supplementary Figure S8). An interaction with cell cycle factors could not be identified at a medium score >0.5.

The results indicate that both *A. thaliana Nse4* genes are highly conserved, and that the corresponding proteins may act in combination with other SMC5/6 complex components, as well as cohesin and condensin. Based on the level of expression, *Nse4A* seems to be the more essential gene, although Nse4B appears to be specialized to act during seed development.

### Selection and Molecular Characterization of *A. thaliana nse4* Mutations and Their Effect on Plant Viability, Fertility, and DNA Damage Repair

From the *A. thaliana* SALK, Syngenta SAIL and GABI-Kat collections, homo- and heterozygous T-DNA insertion mutants were selected for both genes (Figure 1B and Table 1). The presence and positions of corresponding T-DNA insertions were confirmed by PCR using gene-specific and T-DNA specific primers and by sequencing the PCR products (Supplementary Table S2). With exception of line GK-175D11 (intron-insertion in *Nse4B*), all the other T-DNA insertions were found in exons.

**Table 1:** Characterization of the T-DNA insertion mutants of the *A. thaliana Nse4* genes.

Gene symbol	T-DNA mutant	Zygosity	Habit	Pollen fertility (%)	Silique length (mm)	Seeds per silique	Shriveled seeds per silique	% mitotic cells with bridges/fragments	% meiocytes with bridges/fragments		
									Metaphase I	Anaphase I	Anaphase II
Col-wt	–	–	wt	100 (10790)	12.8 (28)	44.5 (1468)	0.7	1.5 (417)	0 (60)	1.2 (67)	0 (23)
*Nse4A*	Salk_057130	He	Smaller	98.2 (3808)	11.3^∗∗^ (24)	30.3^∗∗^ (726)	2.7^∗∗^	11.9^∗∗^ (242)	8.4^∗^ (59)	4.2 (47)	10.0 (21)
	SAIL_71_A08	He									
	GK-768H08	Ho	Smaller	50.2^∗∗^ (6396)	10.0^∗∗^ (25)	21.0^∗∗^ (525)	7.8^∗∗^	25.7^∗∗^ (175)	25.2^∗∗^ (115)	40.4^∗∗^ (114)	47.4^∗∗^ (19)
	GK-768H08 (complemented)	Ho	wt-like	102 (6270)	12.3^∗^ (25)	31.8^∗∗^ (795)	3.4^∗∗^	2.6 (373)	5.0^∗∗^ (121)	19.1^∗∗^ (131)	15.0^∗^ (20)
*Nse4B*	SAIL_296_F02	Ho	wt-like	97.2 (3770)	10.8^∗∗^ (30)	30.5^∗∗^ (916)	1.6	2.1 (278)	0 (59)	5.9 (85)	8.3 (12)
	GK-175D11	Ho	wt-like	64.3^∗∗^ (3206)	11.3^∗∗^ (30)	34.8^∗∗^ (1080)	2.9^∗∗^	1.7 (178)	6.2^∗∗^ (113)	17.8^∗∗^ (106)	0 (18)
*Nse4A/Nse4B*	GK-768H08/SAIL_294_F02	Ho/ho	Smaller	34.8^∗∗^ (4529)	10.2^∗∗^ (30)	17.9^∗∗^ (536)	5.6^∗∗^	28.6^∗∗^ (619)	30.6^∗∗^ (72)	65.0^∗∗^ (172)	50.0^∗∗^ (34)

*The numbers of pollen, siliques, seeds, somatic and meiotic cells analyzed are indicated in parentheses. ^∗∗^*p* < 0.01; ^∗^*p* < 0.05.*

For the *Nse4A* lines Salk_057130 and SAIL_71_A08 only heterozygous mutants could be selected and the progeny segregated into heterozygous and wild-type plants. This indicates the requirement of *Nse4A* for plant viability. The confirmed truncated transcripts downstream outside of the conserved region of the homozygous line GK-768H08 (Figure 1 and Supplementary Figure S9) obviously are able to code at least partially functional proteins. For *Nse4B* two homozygous lines, SAIL_296_F02 and GK-175D11, containing the T-DNA insertion in the second exon and fourth intron, respectively, were identified.

The selected mutants showed a wild-type growth habit, with only a slightly reduced plant size (especially line GK-768H08) compared to wild-type (Figure 2A and Table 1). To combine the mutation effects of *nse4A* and *nse4B*, lines GK-768H08 and SAIL_296_F02 were crossed. The resulting homozygous double mutants showed a further decreased growth. The complementation of the mutation in line GK-768H08 by the genomic wild-type *Nse4A* construct recovered the plant viability.

Thus, the essential character of *Nse4A* becomes confirmed. Although knocking out of *Nse4B* does not induce obvious growth effects, this second *Nse4* homolog is likely not completely free of function.

The selected T-DNA insertion lines were further analyzed more in detail to investigate the influence of the NSE4 proteins on meiosis and fertility. In addition to the reduced plant size, reduced pollen grain number, silique size and seed set together with shriveled seeds were observed in the mutants (Table 1, Figure 2B–D, and Supplementary Figure S10). The aborted seeds might represent the segregating homozygous progeny. The complementation of the mutation in line GK-768H08 by the genomic Nse4A construct recovered pollen fertility and seed setting.

To investigate the DNA damage response of the nse4 mutants compared to wild-type we applied bleomycin at different concentrations in liquid medium to induce DSBs. The treatment clearly impaired the seedling growth of both, the wild-type (Col-0) and the *nse4A* and *nse4B* mutants with increasing bleomycin concentration (Supplementary Figure S11A). To figure out whether the nse4 mutations influence the repair capacity of the plantlets, we performed a similar experiment on solid agar medium plates, and measured the seedling root lengths within 18 days growth (Supplementary Figure S11B). According to a two-way ANOVA a highly significant difference between wild-type and all mutants has been proven regarding the root development without bleomycin treatment. In addition, significantly decreased root growth rates of all three mutants were present after bleomycin application at all concentrations (0.25; 0.5; and 1.0 μg/ml) (Supplementary Figure S11C). These results suggest the involvement of NSE4A and NSE4B in the repair of induced DSBs, and that their mutations may reduce the repair efficiency compared to the wild-type proteins.

### NSE4 Is Essential for Correct Meiosis

**FIGURE 3:**
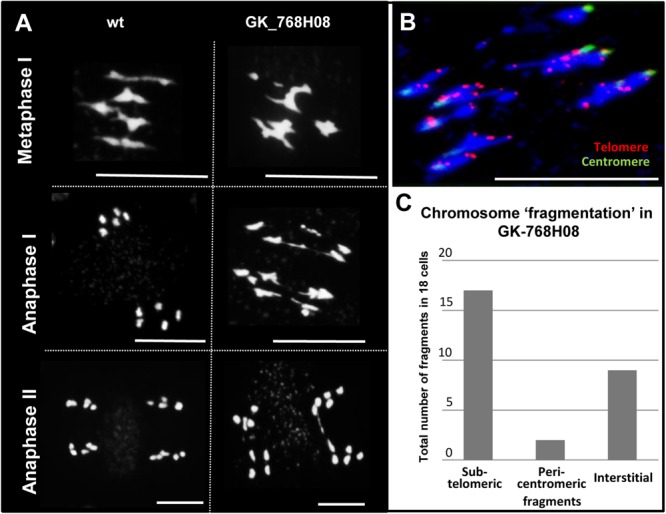
Meiotic defects in the *nse4* mutant GK-768H08. **(A)** Disturbed meiosis (anaphase bridges, fragments) in the *A. thaliana* mutant GK-768H08 compared to wild-type (wt). **(B)** Chromosome fragmentation in GK-768H08 during anaphase I. Telomeres and centromeres were labeled by FISH using centromere- and telomere-specific probes. **(C)** Total number of subtelomeric, pericentromeric, and interstitial chromosome fragments in 18 meiotic cells of the GK-768H08 mutant. Bars = 10 μm.

The reduced number of pollen grains of the *nse4* mutants suggests meiotic disturbances. Therefore, we stained meiocytes by DAPI. During prophase I no apparent alterations were found in the *nse4A* mutant GK-768H08 compared to wild-type. However, anaphase bridges, chromosome fragments and micronuclei appear in later meiotic stages and in tetrad cells, respectively (Figure 3A and Supplementary Figure S12). Micronuclei are a possible product of chromosome fragmentation. In addition to line GK-768H08, all investigated *nse4* mutants showed an increase in meiotic defects, with a clearly increased level in the homozygous GK-768H08/SAIL_294_F02 double mutants. The complementation of the mutation in line GK-768H08 by the genomic *Nse4A* construct abolished mainly the accumulation of meiotic abnormalities (Table 1 and Supplementary Figure S13).

To study the meiotic abnormalities more in detail, FISH experiments using 5S and 45S rDNA probes for chromosome identification were performed (Supplementary Figure S14). The analysis of the *nse4A* mutant GK-768H08 suggests that the occurrence of stretched bivalents, possibly causing chromosome fragments, is not related to specific chromosomes. This indicates that the defects may be induced by disturbing a general meiotic process.

Telomere- and centromere-specific FISH probes were applied to evaluate the proportion of pericentromeric, interstitial and subtelomeric fragments during anaphase I. Most fragments were found to be of subtelomeric origin, followed by interstitial fragments (Figure 3B,C). Obviously, the fragments are the result of a disturbed degree of chromatin condensation along rod bivalents. The increased number of rod bivalents in the mutants seems to be the consequence of a reduced recombination leading to less chiasmata. To test this hypothesis, the chiasma frequency of the *nse4A* mutant GK-768H08 (*n* = 43) was evaluated, and was found to be nearly identical with ∼10.0 chiasmata per diakinesis/metaphase I cell to that of wild-type (Higgins et al., 2004). Thus, the truncation of NSE4A seems not to influence the number of chiasmata.

**FIGURE 4:**
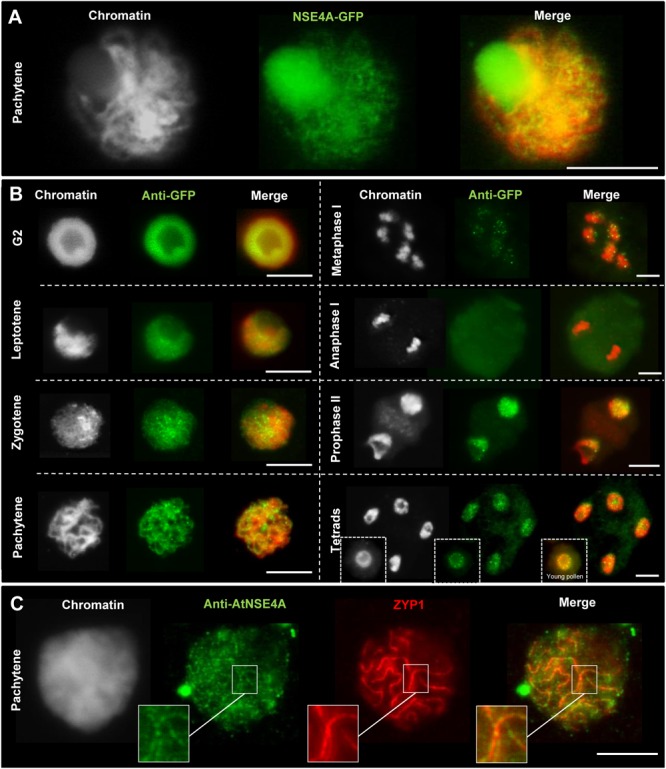
Localization of NSE4A during the meiosis of *A. thaliana* **(A,B)** and the closely related species *B. rapa* **(C)**. **(A)** Line-like NSE4A-GFP signals are detectable in an unfixed meiocyte at pachytene of a transgenic pnse4A::gNse4A::GFP *A. thaliana* plant. **(B)** Dynamics and localization of NSE4A-GFP signals during meiosis of pnse4A::gNse4A::GFP transgenic *A. thaliana* plants, detected by anti-GFP. The NSE4A-GFP signals are detectable in G2, leptotene, zygotene, and pachytene cells. The signals are weak or not visible in condensed metaphase I and anaphase I chromosomes, respectively, but are recovered in prophase II, tetrads and young pollen. **(C)** Anti-AtNSE4A labels the synaptonemal complex of *B. rapa* and colocalizes to ZYP1 during pachytene. Gray color indicates chromatin counterstained with DAPI. Bars = 10 μm.

The occurrence of disturbed meiosis suggests the involvement NSE4 in meiotic processes. Indeed, transgenic *A. thaliana* meiocytes expressing the gNse4A::GFP construct under control of the endogenous promoter showed line-like signals at pachytene, typical for the synaptonemal complex (Figure 4A). In addition, by applying anti-GFP antibodies NSE4A was proven to be present in G2, leptotene, zygotene, and pachytene cells. After mainly disappearing from meta- and anaphase I chromosomes NSE4A recovered in prophase II, tetrads and young pollen (Figure 4B). To confirm the presence of NSE4A in a related species, immunolabeling of *B. rapa* meiocytes with NSE4A-specific antibodies and with ZYP1, the *A. thaliana* transverse filament protein of the synaptonemal complex at pachytene, was performed. The co-localization of both proteins indicated the presence of NSE4A at the synaptonemal complex during pachytene (Figure 4C). The immunolabeling of ZYP1 in pachytene meiocytes of the *nse4A* mutant GK-768H08 indicated that this mutation does not alter the synaptonemal complex structure (Supplementary Figure S15).

We conclude that both NSE4 proteins, but NSE4A again more substantially than NSE4B, are involved in meiotic processes to achieve normal fertility. However, both proteins seem not to influence the frequency of chiasmata, although NSE4A was proven to be present at the synaptonemal complex during prophase I.

### NSE4 Is Present in Interphase Nuclei of Meristem and Differentiated Cells

**FIGURE 5:**
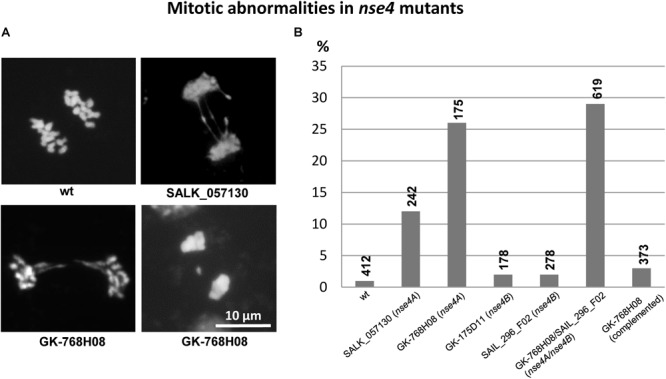
Mitotic defects (anaphase bridges, laggards) in somatic flower bud nuclei of the *A. thaliana nse4* mutants **(A)**. The diagram **(B)** indicates the frequency (%) of abnormalities in the mutants compared to wild-type. The percentage of abnormalities is clearly increased in the *nse4A* mutants SALK_057130 and GK-768H08, as well as in the homozygous double mutant GK-768H08/SAIL_296_F02 representing both *nse4A* and *nse4B* mutations, respectively. The complementation of the mutation in GK-768H08 clearly decreases the number of abnormalities indicating that they are induced by the dysfunction of the Nse4A gene. The numbers of cells analyzed are indicated above the diagram bars.

Similar as during meiosis, abnormalities occur during mitosis in somatic flower bud nuclei of the *A. thaliana nse4* mutants. These mitotic defects occur predominantly in the *nse4A* mutants, and less prominent in the *Nse4B* knock-out mutants (Figure 5).

**FIGURE 6:**
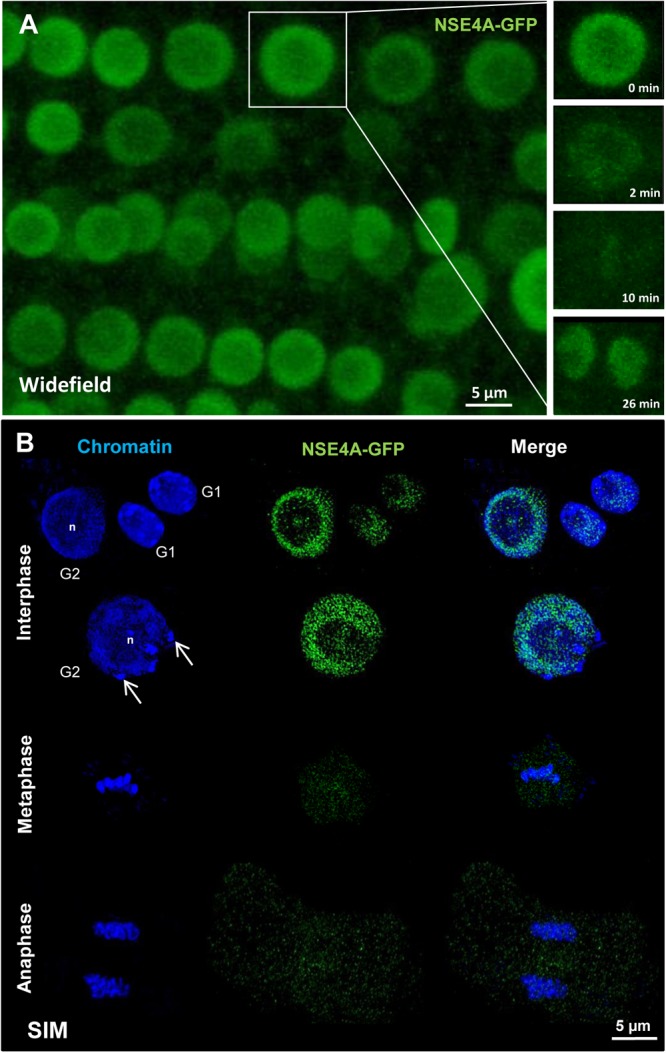
The localization of NSE4A in root meristem cells. **(A)** Global view of a living *A. thaliana* root meristem expressing a genomic *NseA*::GFP construct under the control of the endogenous *Nse4A* promoter. The cell undergoing mitosis (in the rectangle) shows that the nuclear NSE4A-GFP signals are present in interphase (0 min), disappear from the chromosomes during metaphase (2–10 min) and are recovered in telophase at chromatin (26 min). During metaphase a slight cytoplasm labeling is visible. **(B)** The ultrastructural analysis by super-resolution microscopy (SIM) confirms the presence of NSE4A within euchromatin, and indicates its absence from the nucleolus (n) and heterochromatin (chromocenters, arrows) in root meristem G1 and G2 nuclei. During meta- and anaphase NSE4A mainly disappears from the chromosomes, but stays slightly present within the cytoplasm. In young daughter nuclei (G1 phase) NSA4A becomes recovered. The localization of NSE4A-GFP expressed by pnse4A::gNse4A::GFP transgenic *A. thaliana* plants was detected by anti-GFP antibodies in fixed roots.

For live imaging gNSE4A::GFP signals were detected by confocal microscopy in root meristem cells. NSE4A was present in interphase nuclei, disappeared mainly during mitosis from the chromosomes and recovered at telophase at chromatin. Only a slight cytoplasm labeling remained during meta- and anaphase (Figure 6A). To analyze the distribution of NSE4A at the ultrastructural level, fixed interphase nuclei were stained with anti-GFP, and super-resolution microscopy (3D-SIM) has been performed. Thereby, it became obvious that NSE4A is distributed within euchromatin, but absent from nucleoli and chromocenters. During meta- and anaphase only few NSE4A signals were present within cytoplasm, confirming the live cell investigations (Figure 6B).

**FIGURE 7:**
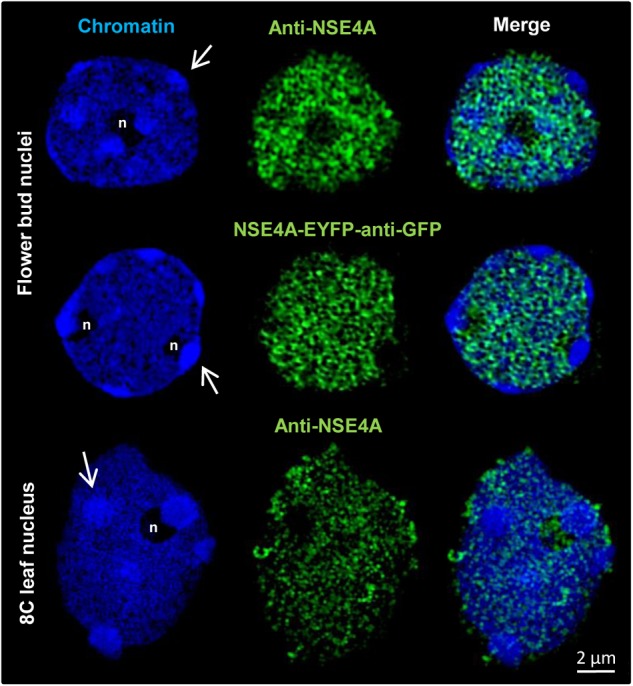
The distribution of NSE4A in differentiated somatic flower bud and 8C leaf interphase nuclei analyzed by 3D-SIM. In agreement, both NSE4A antibodies (anti-NSE4A) and NSE4A-EYFP signals detected by anti-GFP antibodies indicate that NSE4A is distributed within euchromatin, but absent from heterochromatin (DAPI-intense chromocenters, arrows). The NSE4A labeling visible in the merged image of the 8C nucleolus (maximum intensity projection) originates from optical sections outside of the nucleolus.

3D-SIM has also been applied to demonstrate the distribution of NSE4A in differentiated nuclei. Similar as in meristematic tissue, somatic flower bud and 8C leaf interphase nuclei display NSE4A exclusively within euchromatin (Figure 7).

We conclude that, in addition to their meiotic function, NSE4 proteins play also a role in somatic tissue, due to its exclusive presence within the euchromatin of cycling and differentiated interphase nuclei. NSE4A is more prominent than NSE4B also in somatic tissue.

## Discussion

Until now, only few investigations were performed to elucidate the functions of the plant SMC5/6 complexes, their components and interacting factors. We found that *A. thaliana* NSE4 is conserved and multifunctional in distinct chromatin-associated processes during mitosis, meiosis and in differentiated tissue.

### *A. thaliana* Encodes Two Functional and Specialized *Nse4* Variants

Gene duplication has been regarded as a major force in the genome evolution of plants leading to the establishment of new biological functions, such as the production of floral structures, the development of disease resistance, and the adaptation to stress. Duplicated genes can be generated by unequal crossing over, retroposition, chromosomal, and genome duplication (Hurles, 2004; Magadum et al., 2013; Wang and Adams, 2015; Panchy et al., 2016). Compared to other organisms, angiosperms tend to frequent chromosomal duplications and subsequent gene loss (Bowers et al., 2003; Coghlan et al., 2005). In addition, genome duplication in some angiosperms, in particular such with small genomes, seems to be recurrent (Schubert and Vu, 2016). This mediates increased fitness that, however, erodes over time, thus favoring new polyploidization events (Chapman et al., 2006; Innan and Kondrashov, 2010).

The *A. thaliana* genome is a product of a large segment or an entire genome duplication event, which occurred during the early evolution of this species. A comparative sequence analysis against tomato suggests that a first duplication occurred ∼112 million years ago to form a tetraploid (Ku et al., 2000). Altogether, three different duplication events seem to have occurred (Blanc et al., 2003; Bowers et al., 2003). The estimated gene duplication frequency in *A. thaliana* varies from 47% (Blanc and Wolfe, 2004) to 63% (Ambrosino et al., 2016) depending on the methods and parameters used for evaluation.

We confirmed that *A. thaliana* encodes two NSE4 δ-kleisin variants homologous to known NSE4 proteins in other organisms. Both variants show a high and moderate amino acid sequence similarity to plant and non-plant organisms, respectively, and contain a conserved C-terminal domain and a less conserved SMC6 binding motif at its N-terminus (Supplementary Figure S1). Our screening of *Nse4* homologs in other plant species revealed different *Nse4* gene copy numbers, which varied from one in *Eucalyptus grandis* and *Cucumis sativus* up to three copies in *Oryza sativa*. The most other species contain two copies.

Generally, it is not advantageous for species to carry identical functional duplicated genes. Functional and expression divergence are regarded as important mechanisms for the retention of duplicated genes (Semon and Wolfe, 2007). This divergence by mutations results in either pseudogenization (no function anymore), subfunctionalization (partial change of the original function, e.g., tissue specificity) or neofunctionalization (adoption of a new function) (Innan and Kondrashov, 2010; Magadum et al., 2013). The major forces to produce pseudogenes free of function are mutations and deletions, if the gene is not under any selection (Lynch and Conery, 2000). Subfunctionalization appears when the duplicated daughter genes differentiate in some aspects of their functions and adopt a part of the functions of their parental gene (Force et al., 1999). Neofunctionalization leads to evolutionary novel gene functions based on a chance event (mutation) in one of the duplicated genes (Rastogi and Liberles, 2005).

We assume that the two *A. thaliana Nse4* genes are the products of a gene duplication and a subsequently subfunctionalization event (Force et al., 1999). They display a similar sequence and gene structure, but different expression profiles based on our quantitative real-time PCR and *in silico* analyses. While *Nse4A* is expressed in different tissues and developmental stages, *Nse4B* is, in agreement with the findings of Watanabe et al. (2009) almost undetectable in seedlings, rosette leaves, and immature floral buds. Its expression is limited to inflorescence, embryo and endosperm tissues indicating an altered function of NSE4B during seed development, which apparently can be substituted, at least in part, by other cellular components in *nse4B* mutants.

The results suggest that *Nse4A* and *Nse4B* became specialized during evolution, possibly based on a process named duplication-degeneration-complementation. This process comprises complementary degenerative mutations in different regulatory elements of duplicated genes which can facilitate the preservation of both duplicates. Thus, the process provokes that degenerative mutations in regulatory elements can increase the probability of duplicate gene preservation, and that the ancestral gene function is rather portioned out to the daughter genes, instead of developing new functions (Force et al., 1999, 2005; Feder, 2007). Based on such a process *Nse4A* may have maintained its multiple functions in the various tissues like the ancestral gene before duplication. Instead, *Nse4B* achieved specialized functions during seed development as a paralog of *Nse4A*.

Interestingly, in other plant and non-plant organisms, the expression patterns differ also between the two *Nse4* variants suggesting a gene subfunctionalization process. In *Z. mays*, two *Nse4* homologs exist. One of them is highly expressed across different tissues, whereas its paralog is expressed in seed tissues and only weakly or not at all in other tissues^15^.

The finding that NSE4A and NSE4B contain specific degradation motifs, and SUMOylation sites in addition to the common ones suggests, that the amount of both proteins in different tissues of *A. thaliana* might be differentially regulated not only at the level of transcription, but also at the protein level. The presence of some specific SUMOylation sites in both proteins might suggest their different regulation during the cell cycle and development, since SUMOylation plays an important role in these processes (Park et al., 2011).

The human genome encodes also two *Nse4* gene variants which are ∼50% identical depending on the isoform analyzed^16^. Also in human one *Nse4* gene is expressed in different somatic tissues, whereas the second one is expressed exclusively in generative tissues (Båvner et al., 2005; Taylor et al., 2008). NSE1, NSE3, and NSE4 can form a sub-complex associated to the SMC5–SMC6 head domain binding sites in yeast (Sergeant et al., 2005; Pebernard et al., 2008; Hudson et al., 2011; Kozakova et al., 2015). Thus, the finding of Li et al. (2017) that NSE1 and NSE3 of *A. thaliana* are required for early embryo and seedling development, confirms our observation that also NSE4 is expressed in these tissues.

We conclude that *A. thaliana* comprises two conserved *Nse4* genes, which may have undergone subfunctionalization and can be regarded as functional paralogs.

### NSE4 Acts in Meristematic and Differentiated Interphase Nuclei

In interphase nuclei, SMC complexes organize chromatin into a higher order and are responsible for the dynamics of chromatin. They regulate replication, segregation, repair, and transcription (Carter and Sjögren, 2012). The composition of the *A. thaliana* SMC5/6 complex (Figure 1) was predicted based on data available for yeast and animals. Our *in silico* generated protein-protein interaction network (Supplementary Figure S8) confirmed this prediction. In a recent publication of Diaz et al. (2019) interactions of both NSE4A and NSE4B with NSE3 and SMC5 were confirmed experimentally. However, the interactions of NSE4A and NSE4B with SMC6A, SMC6B, and NSE1 could not be detected. The similar composition and structure of the SMC5/6 complex compared to cohesin and condensin and the ability to bind to DNA (Alt et al., 2017) suggests that all SMC complexes may share a similar topological distribution in interphase chromatin. This idea is supported by the observation that SMC5/6 binds to DNA also via the kleisin-kite subcomplex NSE1-NSE3-NSE4 (Zabrady et al., 2016), similar as the condensin binding to DNA via the kleisin-hawk subcomplex (Kschonsak et al., 2017). Using the protein-protein interaction database STRING, we can also predict interactions of the SMC5/6 complex components with cohesin and condensin proteins (Supplementary Figure S8).

Interestingly, in *Drosophila* SMC5/6 is enriched in heterochromatin and required to prevent abnormal homologous recombination repair (Chiolo et al., 2011). Instead, we found *A. thaliana* NSE4 distributed exclusively within the euchromatin of differentiated and meristematic interphase nuclei, similar as described for components of the *A. thaliana* cohesin and condensin complex (Schubert et al., 2013). This suggests a similar role of these proteins in interphase. Interestingly, also transiently expressed *A. thaliana* NSE1 and NSE3 (components of the NSE1-NSE3-NSE4 sub-complex) proteins were shown to be present in tobacco leave nuclei (Li et al., 2017).

Our finding that NSE4 localized in relaxed “open” euchromatin known to be transcriptionally active (Li et al., 2007) and not in “closed” highly condensed heterochromatin suggest a role of these proteins in transcriptional regulation. This idea is supported by the observations that human NSE4 is present in interphase chromatin but absent from nucleoli (Taylor et al., 2001), and that it is acting as a transcriptional suppressor (Båvner et al., 2005). Based on Hi-C investigations Lieberman-Aiden et al. (2009) suggested the organization of human interphase chromatin in open and closed regions. SMC complexes may be involved in the control of the extrusion or drawing back of transcriptional loops.

RNA polymerase II molecules, similar as SMC proteins, are exclusively distributed within the euchromatin of interphase nuclei. SMCs may contribute to transfer chromatin into a transcriptional active form (“open” euchromatin), to be accessible for RNA polymerase II performing transcription (Schubert, 2014; Schubert and Weisshart, 2015).

While *A. thaliana* NSE4 was present in interphase nuclei, it mainly disappeared from the chromosomes during mitosis. In non-plant organisms, the localization of SMC5/6 is contradictory. Similar as *A. thaliana* NSE4, human SMC5 and SMC6 are present in interphase nuclei, dissociate from chromosomes at mitosis and then, co-localizes again with chromatin at the G1 phase. In addition, a cytoplasm staining was detectable (Taylor et al., 2001; Gallego-Paez et al., 2014; Verver et al., 2014). In contrast, mouse SMC6 co-localized with centromeric heterochromatin during interphase as well as in mitosis, and with the chromatid axes of somatic metaphase chromosomes (Gomez et al., 2013). In budding yeast SMC6, NSE1, SMC5, and NSE4 all interact with the centromeric regions in G2/M phase-arrested cells (Lindroos et al., 2006). In fission yeast SMC5/6 complexes combine recombination repair with kinetochore protein regulation (Yong-Gonzales et al., 2012), and NSE4 is required for the metaphase to anaphase transition (Hu et al., 2005). These observations indicate a role of SMC5/6 in the maintenance of centromere and higher order metaphase chromosome structure in these organisms.

Similar as described for *A. thaliana nse1* and *nse3* (Li et al., 2017) we found mitotic defects (anaphase bridges, chromosome fragmentation, micronuclei formation) in our *nse4* mutants. Somatic anaphase bridges and micronuclei were also documented in human and yeast SMC5/6 subunit depleted cells (Pebernard et al., 2004; Bermúdez-López et al., 2010; Gallego-Paez et al., 2014). Importantly, micronuclei and chromatin phenotypes are associated with *nse3* mutations in human LICS syndrome cells, exhibiting a reduced level of SMC5/6 complexes (van der Crabben et al., 2016). SMC5/6 is essential in DNA replication by preventing the formation of supercoiled DNA and/or sister chromatid intertwining (Jeppsson et al., 2014a; Verver et al., 2016; Diaz and Pecinka, 2018) which may cause anaphase bridges and chromosome missegregation. These mitotic defects may originate from disturbed SMC5/6 complex functions in G2 and prophase. Although we document the absence of NSE4A from mitotic chromosomes, it seems that the *A. thaliana* SMC5/6 complex is involved in the topological organization of chromatin during mitotic chromosome condensation and decondensation. The mitotic defects in our *nse4A* mutants might be explained by an incorrect SMC5/6 ring formation which is essential for its proper function. Thus, the lack or truncation of NSE4 may result in an impaired catalytic and/or topological SMC5/6 complex function.

The catalytic activity of SMC5/6 is provided by the E3 SUMO-protein ligase NSE2 (Fernandez-Capetillo, 2016), and is essential to globally facilitate the resolution of intermediates during homologous sister chromatid recombination (Bermúdez-López et al., 2010; Chavez et al., 2010), which unresolved may also cause anaphase bridges (Chan et al., 2018).

Mitotic defects may also be induced by an impaired topological function of SMC5/6. Similar as the other SMC complexes, SMC5/6 is an ATP-dependent intermolecular DNA linker (Kanno et al., 2015). Hence, it is not astonishing that the inhibition of SMC5/6 has also been linked to sister chromatid cohesion defects in *Arabidopsis*, chicken and human cells (Watanabe et al., 2009;Stephan et al., 2011; Gallego-Paez et al., 2014).

The idea that SMC5/6 is involved in organizing chromatin topology is also supported by the finding that human SMC5/6 is required for regulating topoisomerase IIα and condensin localization on replicated chromatids in cells during mitosis, thus ensuring correct chromosome morphology and segregation (Gallego-Paez et al., 2014). By introducing DSBs topoisomerase II resolves DNA topological constraints and decatenates dsDNA to reduce supercoiling (Nitiss, 2009).

We found a slight *A. thaliana* NSE4A labeling within the cytoplasm during meta- and anaphase. Mitotically released SMC5/6 complexes might be engaged in a NSE2 mediated signaling pathway in the cytoplasm to regulate the mitotic cell cycle in plant and non-plant organisms (Huang et al., 2009; Ishida et al., 2009; Park et al., 2011; Mukhopadhyay and Dasso, 2017). It has also been reported that yeast SMC5 can bind and stabilize microtubules to realize proper spindle structures and mitotic chromosome segregation (Laflamme et al., 2014).

We applied bleomycin to induce DNA DSBs and found that both *nse4A* and *nse4B* mutations cause a reduced DNA repair efficiency compared to wild-type. In contrast, although Diaz et al. (2019) report an effect of NSE4A on somatic DNA damage repair, they did not prove an influence of bleomycin treatment, possibly due to the significantly lower concentration applied. We conclude, that the presence of *A. thaliana* NSE4A in euchromatin and the disturbance of mitotic divisions by NSE4 mutations indicate an involvement of this SMC5/6 complex component in interphase chromatin organization of differentiated and cycling somatic nuclei. Thus, NSE4 seems to be important for transcriptional regulation, as well as for correct DNA repair and replication by preventing chromatin supercoiling and resolving sister chromatid intertwining to realize correct mitosis.

### NSE4 Acts During Meiosis

The mutants of both *Nse4A* and *Nse4B* display reduced silique length, pollen and seed number. This fertility reduction seems to be related to the observed meiotic abnormalities, such as anaphase bridges, lagging chromosomes, chromosome fragmentation and micronuclei formation. Previously, a decreased seed set has also been observed in other *A. thaliana* SMC5/6 subunit mutants, such as *smc6B* (Watanabe et al., 2009), *nse1, nse3* (Li et al., 2017), and *nse2* (Ishida et al., 2012).

Similar abnormalities in meiosis as found in mitosis may be based on similar disturbed molecular mechanisms. Somatic anaphase bridges may originate from unresolved sister chromatid intertwining, whereas bridges between bivalents can also be caused by aberrant recombination intermediates between homologous chromosomes. as found in yeast (Copsey et al., 2013; Xaver et al., 2013). DSBs induce the activation of the SMC5/6 complex by auto-SUMOylation, thus activating the yeast SGS1-TOP3-RMI (STR) complex. STR is engaged in a proper DSB repair and crossover pathway during homologous recombination in somatic cells (Bermudez-Lopez et al., 2016; Bermúdez-López and Aragon, 2017). A critical role for STR was also demonstrated in meiosis of yeast (Jessop et al., 2006; Oh et al., 2007). In *A. thaliana*, a similar mechanism might exist, as suggested by the presence of the yeast STR complex ortholog AtRTR (RECQ4A-TOP3α-RMI). The RTR complex is responsible for genome stability and the dissolution of recombination intermediates in meiosis (Knoll et al., 2014). The involvement of SMC5/6 in preventing aberrant meiotic recombination intermediates was also found in non-plant organisms such as yeast (Farmer et al., 2011) and worm (Hong et al., 2016).

We describe that *A. thaliana* NSE4A does not influence the number of chiasmata. Also data from yeast (Farmer et al., 2011; Lilienthal et al., 2013) and worm (Bickel et al., 2010) indicate that SMC5/6 functions during joint-molecule resolution without influencing crossover formation, suggesting that SMC5/6 is primarily involved in resolving the intermediates of sister chromatid recombination rather than of inter-homolog recombination. On the other hand, a linkage between SMC5/6 and crossover formation cannot be excluded, because in rye the colocalization of human enhancer of invasion-10 (HEI10) and *A. thaliana* NSE4A homologs has been proven (Hesse et al., 2019). HEI10 is a member of the ZMM (ZIP1/ZIP2/ZIP3/ZIP4, MSH4/MSH5, and MER3) protein family essential for meiotic recombination in different eukaryotes (Toby et al., 2003; Whitby, 2005; Osman et al., 2011; Chelysheva et al., 2012; Wang et al., 2012).

The observed meiotic anaphase bridges and the formation of chromosome fragments may be caused not only by a disturbed recombination intermediate resolution. As observed in our *nse4A* mutant, rod bivalents may be extensively stretched. Such a chromatin elongation may also be due to impaired chromatin condensation. Condensin I and II are essential factors involved in correct chromatin condensation and chromosome segregation during mitosis and meiosis. They localize at the metaphase chromatid axes and thus, form a dynamic chromosome scaffold (Maeshima and Laemmli, 2003; Chan et al., 2004; Cuylen and Haering, 2011; Houlard et al., 2015; Kinoshita and Hirano, 2017; Kakui and Uhlmann, 2018; Paul et al., 2018).

Several publications indicate that there is a functional relationship between condensin and SMC5/6. In worms inter-homolog bridges were described in *smc5* mutants inducing an irregular condensin distribution along bivalents, as well as chromosome condensation defects (Hong et al., 2016). Also in mouse oocytes SMC5/6 was shown to assist condensin functions during meiosis I (Houlard et al., 2015; Hwang et al., 2017) and in embryonic stem cells during mitosis (Pryzhkova and Jordan, 2016). Furthermore, in human RPE-1 cells the RNAi-mediated depletion of SMC5 and SMC6 resulted in defective axial localization of condensin in mitosis (Gallego-Paez et al., 2014).

In non-plant organisms, such as worm, mouse and human (Taylor et al., 2001; Bickel et al., 2010; Gomez et al., 2013; Verver et al., 2013, 2014) SMC5/6 subunits were observed at the synaptonemal complex. We found a chromatin-specific localization of *A. thaliana* NSE4A in premeiotic G2, in prophase I, II and in tetrad cells. At prophase I of rye (Hesse et al., 2019), *A. thaliana* and *B. rapa*, NSE4A creates line-like structures colocalizing to ZYP1, a central element of the synaptonemal complex. This suggests that NSE4 might also be involved in the synaptonemal complex formation of plants. Thus, impaired NSE4 in the *nse4* mutants could be another reason for the observed meiotic defects and reduced fertility.

Our data indicate a role of plant NSE4A in proper meiotic chromosome segregation via realizing correct chromatin condensation, recombination intermediate resolution and synapsis.

## Data Availability

Publicly available datasets were analyzed in this study. This data can be found here: https://myhits.isb-sib.ch/cgi-bin/motif_scan.

## Author Contributions

VS, MZ, UC, and AH conceived the study and designed the experiments. AH and VS contributed equally to supervise the project. MZ, KZ, UC, SH, IL, MM, and VS performed the experiments. AM performed the statistics. VS and MZ wrote the manuscript. All authors read and approved the final manuscript.

## Conflict of Interest Statement

The authors declare that the research was conducted in the absence of any commercial or financial relationships that could be construed as a potential conflict of interest.

## Footnotes

1 http://signal.salk.edu/cgi-bin/tdnaexpress

2 http://www.gabi-kat.de/

3 http://nasc.nott.ac.uk

4 ncbi.nlm.nih.gov

5 pfam.sanger.ac.uk

6 https://www.ncbi.nlm.nih.gov/Structure/cdd/wrpsb.cgi

7 mrbayes.sourceforge.net/

8 www.ebi.ac.uk/Tools/msa/clustalo/

9 https://www.uniprot.org/uniprot/Q9NXX6

10 https://www.uniprot.org/uniprot/Q8N140

11 https://www.ebi.ac.uk/interpro/entry/IPR014854

12 https://myhits.isb-sib.ch/cgi-bin/motif_scan

13 http://elm.eu.org/

14 http://string-db.org/

15 https://www.maizegdb.org/gene_center/gene/GRMZM2G026802

16 https://www.uniprot.org/uniprot

## Supplementary Material

FIGURE S1 Amino acids sequence alignment between full-length NSE4A and NSE4B. The alignment was performed by the Clustal Omega 2.1 software (https://www.ebi.ac.uk/Tools/msa/clustalo/). ^∗^, Identical amino acids; :, similar amino acids; –, missing amino acids. The conserved functional protein domains were predicted using the Motif Scan (https://myhits.isb-sib.ch/cgi-bin/motif_scan) and Eukaryotic Linear Motif (http://elm.eu.org/) resources. The putative SMC6-binding domain and the conserved C-terminal NSE4_C domain are highlighted in turquoise and yellow, respectively. The amino acids of putative degradation regions and SUMOlisation sites are labeled in red and in green, respectively. The amino acids “VKPE” marked in blue are a SUMOlisation site overlapping with the amino acids “KPGAGVKPE” of a putative degradation site. The region used to produce recombinant anti-NSE4A antibodies is underlined. NSE4A and NSE4B share 67.7% sequence identity.

FIGURE S2 Proof of the NSE4A antibody specificity. **(A)** Western blots showing the correct size (∼28 kDa) of the expressed recombinant NSE4A protein. The purified NSE4A proteins (1: 1.4 μg, 2: 1.4 μg, 3: 0.7 μg) were separated on a 10% SDS-PAA gel, stained with Coomassie Blue (1) or electro-transferred and visualized after Western Blot by anti His-Tag antibodies (2), or anti T7 antibodies via anti mouse-POD conjugate by ECL detection (3) (Conrad et al., 1997). **(B)** Competitive ELISA showing the specific NSE4A antibody binding behavior. The binding of antibodies to the solid phase adsorbed antigens was specifically inhibited in a concentration-dependent manner by competition with different concentrations of soluble NSE4A to detect at which concentrations of soluble antigens a strong competition can be achieved. A nearly complete inhibition was observed at 200 nmol. **(C)** The incubation of the anti-NSE4A antibodies with recombinant NSE4A proteins prior immunostaining resulted in the signal reduction in *A. thaliana* 8C leaf interphase nuclei.

FIGURE S3 Amino acids sequence alignment between NSE4A of *A. thaliana* and two putative NSE4A proteins (XP_009144924 and XP_009147782) of *B. rapa*. The alignment was performed by the Clustal Omega 2.1 software (https://www.ebi.ac.uk/Tools/msa/clustalo/). ^∗^, Identical amino acids; :, similar amino acids; –, missing amino acids. The conserved C-terminal NSE4 domain is highlighted in yellow. NSE4A shows 77.7 and 80.1% identity to XP_009144924 and XP_009147782, respectively.

FIGURE S4 Phylogenetic relationships of the *A. thaliana* NSE4A and NSE4B proteins. **(A)** Percentage of plant protein identities compared to *A. thaliana* NSE4A. **(B)** The phylogenic NSE4 tree was reconstructed based on full-length protein sequences of known putative NSE4 orthologs of plants available at NCBI https://www.ncbi.nlm.nih.gov/. *Physcomitrella* was defined as outgroup. Eudicot-derived sequences are given in blue, monocots in red. Numbers at nodes provide Bayesian posterior probabilities indicating clade support. The scale bar represents the average number of amino acid substitutions per site.

FIGURE S5 *In silico* analysis of the relative *in silico* expression level of the *Nse4A* and *Nse4B* genes during plant development compared to other SMC5/6 subunit genes (genevestigator.com). Stages 1–3 indicate young seedlings and rosettes; 4–6 developed rosettes, bolting and young flowers; 7–9 mature flowers, siliques, and seed stages.

FIGURE S6 *In silico* analysis of the relative expression level of *Nse4A* (blue) and *Nse4B* (red) in ten anatomical parts from 431 individual sequencing samples of *A. thaliana* (Col-0; AT_mRNASeq_ARABI_GL-0 databases https://genevestigator.com/). Standard deviation is indicated.

FIGURE S7 Relative expression of *Nse4A* in different *A. thaliana* tissues compared to the reference genes *Pp2A* **(A)** and *Rhip1* **(B)**. The experiments were performed by quantitative real-time PCR. Three technical and biological replicates were realized. Standard deviation is indicated.

FIGURE S8 Both *A. thaliana* NSE4 proteins interact potentially with other SMC5/6 components **(A)**, as well as with cohesin and condensin complex subunits **(B)**. The protein-protein interaction network was generated based on a STRING program (http://string-db.org/) analysis at scores >0.95 and >0.70, respectively. The black lines in between the proteins indicate the supporting evidence from experimental data available from different species. Interactions confirmed experimentally for *A. thaliana* by Diaz et al. (2019) are indicated by red lines.

FIGURE S9 RT-PCR-based confirmation of the NSE4A truncation in the T-DNA mutant line GK-768H08. **(A)** Schemata of the *nse4A* gene structure and length of PCR products in wt and the mutant. **(B)** Electrophoresis indicates the absence of the full-length Nse4A transcript in line GK-768H08 compared to wt.

FIGURE S10 *nse4* mutations result in reduced fertility (% pollen per anther). Only the SALK_057130 and SAIL_296_F02 T-DNA insertion lines do not show a significantly decreased fertility compared to wild-type (wt). In the complemented line GK-768H08 the complete wild-type fertility is recovered. The numbers of evaluated pollen grains are indicated above the diagram bars. Standard deviation is indicated.

FIGURE S11 DNA damage response of the *nse4* mutants compared to wild-type (Col-0) after bleomycin application at different concentrations (μg/ml) to induce DSBs. **(A)** The increasing bleomycin concentration clearly impairs the plantlet growth in liquid medium. **(B)** The bleomycin treatment (here shown, e.g., 0.5 μg/ml; right) also reduces the root growth of the plantlets on agar plates in comparison to the untreated control (left), as indicated here on 14-day-old plantlets. **(C)** Diagrams C_1_–C_4_ show the root development on agar plates of Col-0 compared to the mutants at different bleomycin concentrations. All mutants show a significantly decreased root length growth relative to Col-0 according to a two-way ANOVA. Diagram C_5_ demonstrates the negative influence of the increasing bleomycin concentration on the root development. Diagram C_6_ demonstrates that compared to Col-0 all mutants are significantly stronger negatively influenced at all bleomycin concentrations (ANOVA: *P* < 0.05). Standard errors of mean are indicated in diagrams C_1_–C_5_.

FIGURE S12 No abnormalities during prophase I **(A)**, but micronuclei appear in prophase II and tetrads **(B)** of the *nse4A* mutant GK-768H08 compared to wt. The micronuclei (arrows) may originate from chromatin bridges and fragment formation during metaphase I, anaphase I and II (see Figure 3). Chromatin was stained with DAPI.

FIGURE S13 Meiotic abnormalities (% fragments, anaphase bridges) in *nse4* mutants during different meiotic stages compared to wt. The complementation in line GK-768H08 partially recovers the normal meiotic wt behavior. The numbers of evaluated meiocytes are indicated above the diagram bars.

FIGURE S14 Chromosomal abnormalities during metaphase I in the *nse4A* mutant GK-768H08 compared to wt. **(A)** Karyotype of *A. thaliana* indicating the chromosomal positions of 5S rDNA (red) and 45S rDNA (green). **(B)** The chromosomal distribution of the 5S and 45S rDNA repeats allows the identification of the different *A. thaliana* bivalents. Due to stretched rod bivalents, chromatin fragments (arrows) occur at chromosomes 4 **(C)** and 2 **(D)**.

FIGURE S15 The *nse4A* mutant GK-768H08 shows a wt-like localization of the central synaptonmal complex protein ZYP1 at pachytene. Chromatin was counterstained with DAPI (blue).

TABLE S1 Primers used to identify the T-DNA insertion alleles.

TABLE S2 Sequences of the left border junctions of the T-DNA insertion lines. The red letters represent the sequence derived from the T-DNA, and their position in each of the sequences reflects the orientation of the inserted T-DNA.

TABLE S3 Quantitative real-time RT-PCR primers used to amplify transcripts.

TABLE S4 Primers used to clone the *Nse4A* genes, to produce clones for recombinant protein expression, and for the transcript analyses of the mutants and transgenic lines.

TABLE S5 *Arabidopsis thaliana* NSE4 protein sequence identities (%) compared to orthologs of non-plant organisms. The matrix was generated by the Clustal Omega 2.1 software.

TABLE S6 Genes showing high co-expression with Nse4A predicted from 18 different anatomical tissues. The data were obtained from the AT_mRNASeq_ARABI_GL-0 database of https://genevestigator.com. Scores indicate the level of correlation of expression in different anatomical samples. Bold gene names indicate meiosis- or chromatin-related genes.

## Abbreviations

aa: amino acids
ANOVA: analysis of variance
dsDNA: double strand DNA
DSB: double strand break
FISH: fluorescence *in situ* hybridization
GFP: green fluorescent protein
PCR: polymerase chain reaction
PMC: pollen mother cell
PPT: phosphinothricin
SIM: structured illumination microscopy
