# Supplementary figures and images for: Arabidopsis NSE4 Proteins Act in Somatic Nuclei and Meiosis to Ensure Plant Viability and Fertility
Source: Front Plant Sci. 2019 Jun 20;10:774. doi: 10.3389/fpls.2019.00774 (PMC6596448; doi:10.3389/fpls.2019.00774)

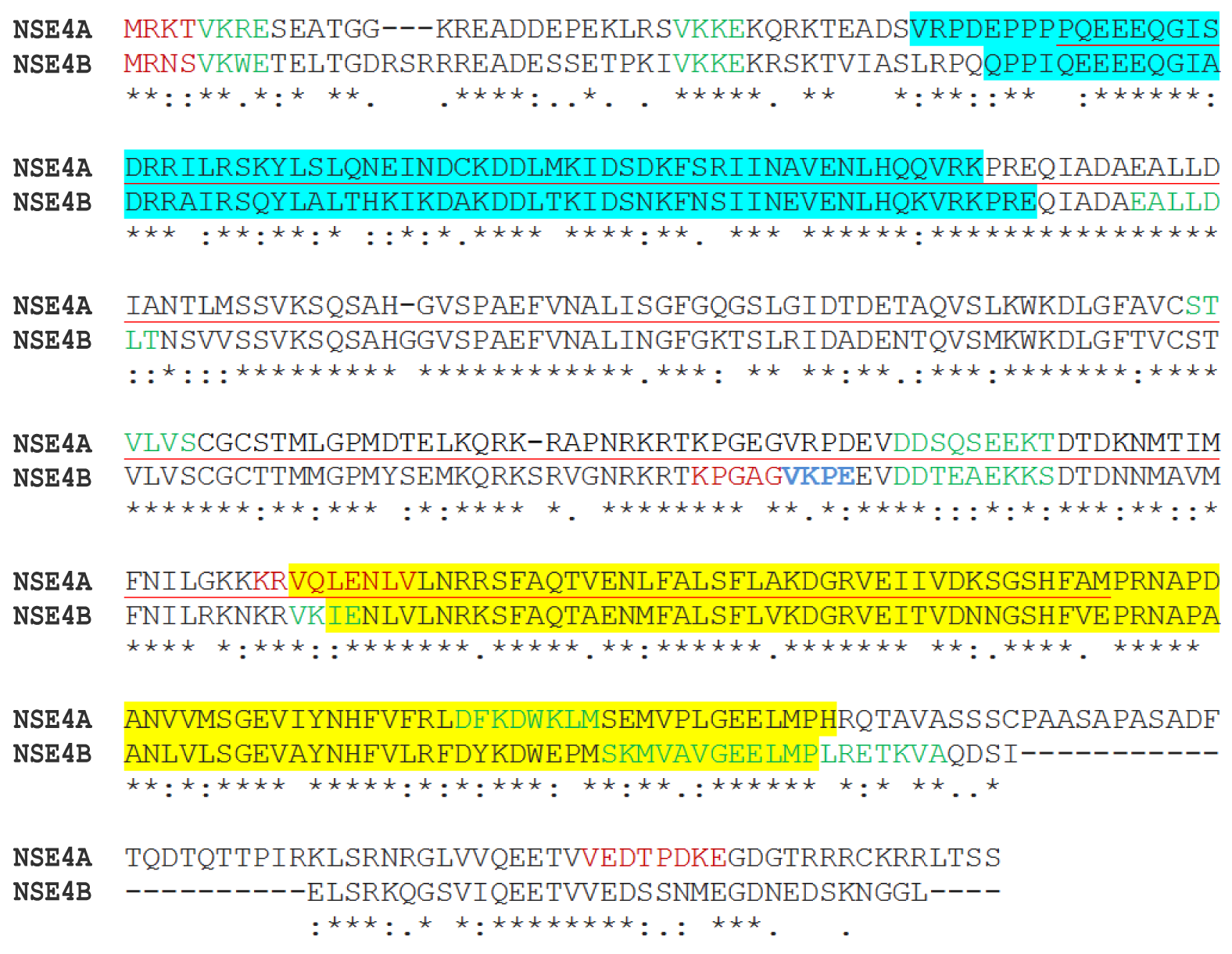

Supplement: FIGURE S1 — Amino acids sequence alignment between full-length NSE4A and NSE4B. The alignment was performed by the Clustal Omega 2.1 software (https://www.ebi.ac.uk/Tools/msa/clustalo/). ∗, Identical amino acids; :, similar amino acids; –, missing amino acids. The conserved functional protein domains were predicted using the Motif Scan (https://myhits.isb-sib.ch/cgi-bin/motif_scan) and Eukaryotic Linear Motif (http://elm.eu.org/) resources. The putative SMC6-binding domain and the conserved C-terminal NSE4_C domain are highlighted in turquoise and yellow, respectively. The amino acids of putative degradation regions and SUMOlisation sites are labeled in red and in green, respectively. The amino acids “VKPE” marked in blue are a SUMOlisation site overlapping with the amino acids “KPGAGVKPE” of a putative degradation site. The region used to produce recombinant anti-NSE4A antibodies is underlined. NSE4A and NSE4B share 67.7% sequence identity. [file Presentation_1.zip › SupplFig1.tif]

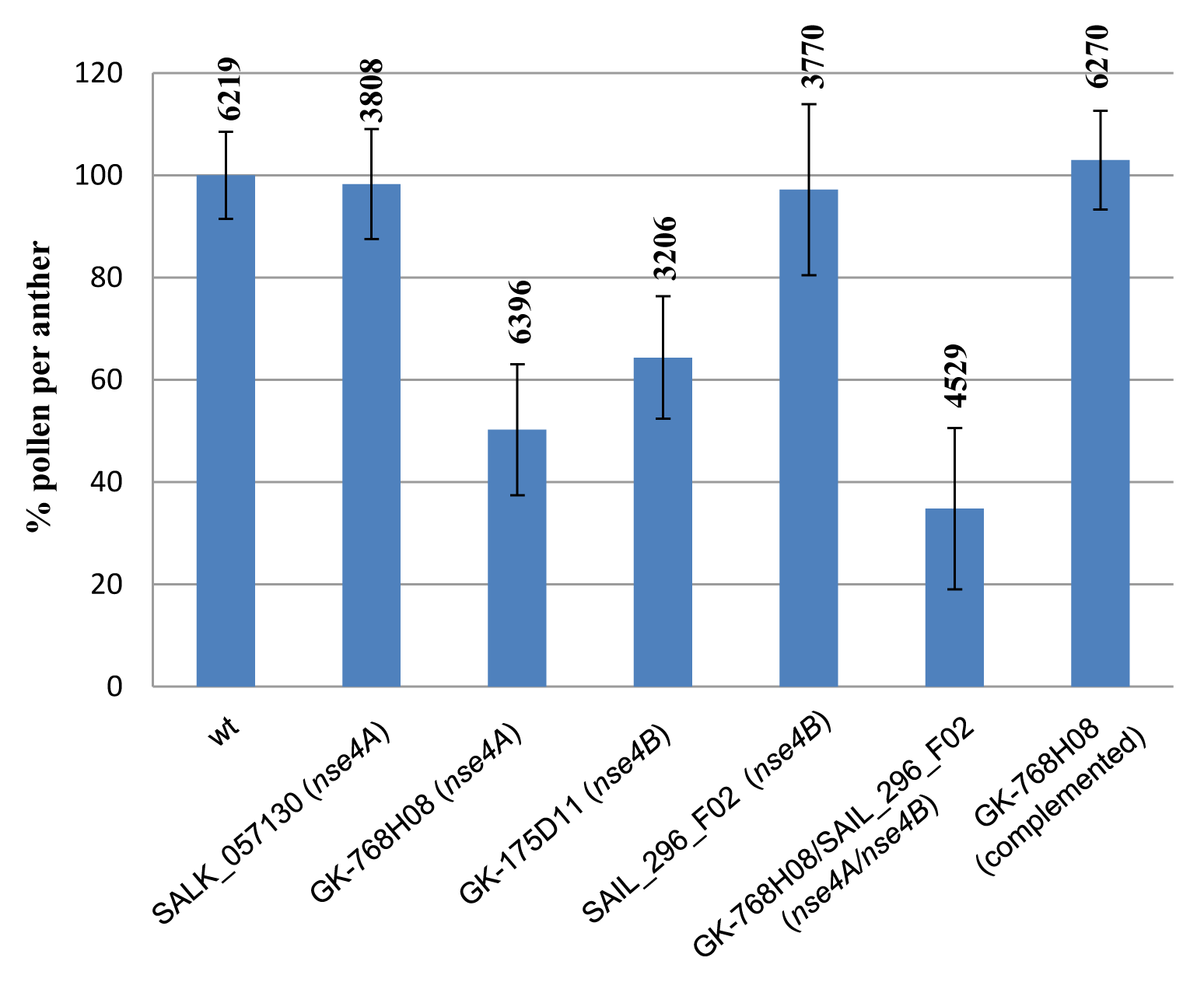

Supplement: FIGURE S1 — Amino acids sequence alignment between full-length NSE4A and NSE4B. The alignment was performed by the Clustal Omega 2.1 software (https://www.ebi.ac.uk/Tools/msa/clustalo/). ∗, Identical amino acids; :, similar amino acids; –, missing amino acids. The conserved functional protein domains were predicted using the Motif Scan (https://myhits.isb-sib.ch/cgi-bin/motif_scan) and Eukaryotic Linear Motif (http://elm.eu.org/) resources. The putative SMC6-binding domain and the conserved C-terminal NSE4_C domain are highlighted in turquoise and yellow, respectively. The amino acids of putative degradation regions and SUMOlisation sites are labeled in red and in green, respectively. The amino acids “VKPE” marked in blue are a SUMOlisation site overlapping with the amino acids “KPGAGVKPE” of a putative degradation site. The region used to produce recombinant anti-NSE4A antibodies is underlined. NSE4A and NSE4B share 67.7% sequence identity. [file Presentation_1.zip › SupplFig10.tif]

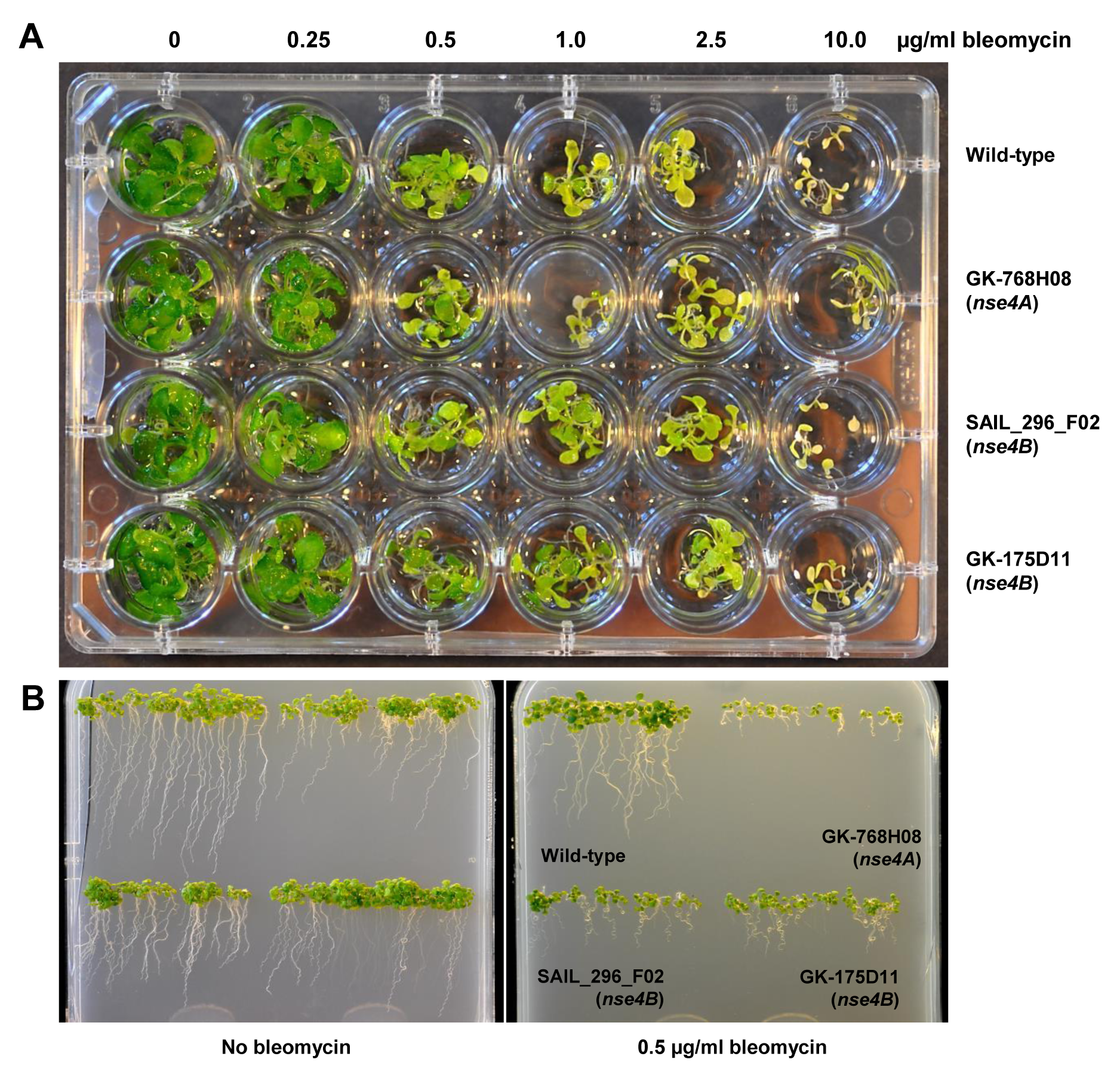

Supplement: FIGURE S1 — Amino acids sequence alignment between full-length NSE4A and NSE4B. The alignment was performed by the Clustal Omega 2.1 software (https://www.ebi.ac.uk/Tools/msa/clustalo/). ∗, Identical amino acids; :, similar amino acids; –, missing amino acids. The conserved functional protein domains were predicted using the Motif Scan (https://myhits.isb-sib.ch/cgi-bin/motif_scan) and Eukaryotic Linear Motif (http://elm.eu.org/) resources. The putative SMC6-binding domain and the conserved C-terminal NSE4_C domain are highlighted in turquoise and yellow, respectively. The amino acids of putative degradation regions and SUMOlisation sites are labeled in red and in green, respectively. The amino acids “VKPE” marked in blue are a SUMOlisation site overlapping with the amino acids “KPGAGVKPE” of a putative degradation site. The region used to produce recombinant anti-NSE4A antibodies is underlined. NSE4A and NSE4B share 67.7% sequence identity. [file Presentation_1.zip › SupplFig11.tif]

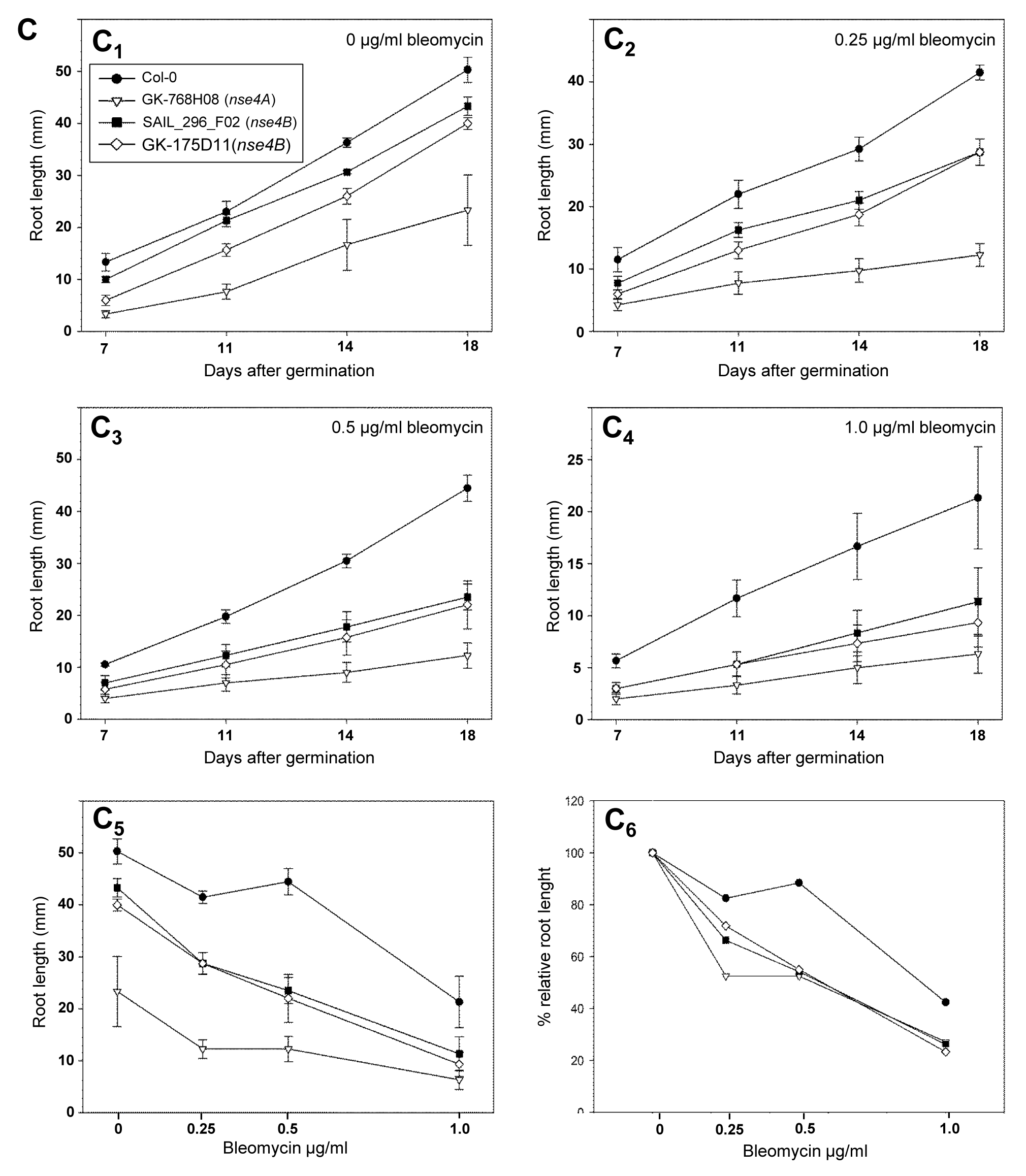

Supplement: FIGURE S1 — Amino acids sequence alignment between full-length NSE4A and NSE4B. The alignment was performed by the Clustal Omega 2.1 software (https://www.ebi.ac.uk/Tools/msa/clustalo/). ∗, Identical amino acids; :, similar amino acids; –, missing amino acids. The conserved functional protein domains were predicted using the Motif Scan (https://myhits.isb-sib.ch/cgi-bin/motif_scan) and Eukaryotic Linear Motif (http://elm.eu.org/) resources. The putative SMC6-binding domain and the conserved C-terminal NSE4_C domain are highlighted in turquoise and yellow, respectively. The amino acids of putative degradation regions and SUMOlisation sites are labeled in red and in green, respectively. The amino acids “VKPE” marked in blue are a SUMOlisation site overlapping with the amino acids “KPGAGVKPE” of a putative degradation site. The region used to produce recombinant anti-NSE4A antibodies is underlined. NSE4A and NSE4B share 67.7% sequence identity. [file Presentation_1.zip › SupplFig11continued.tif]

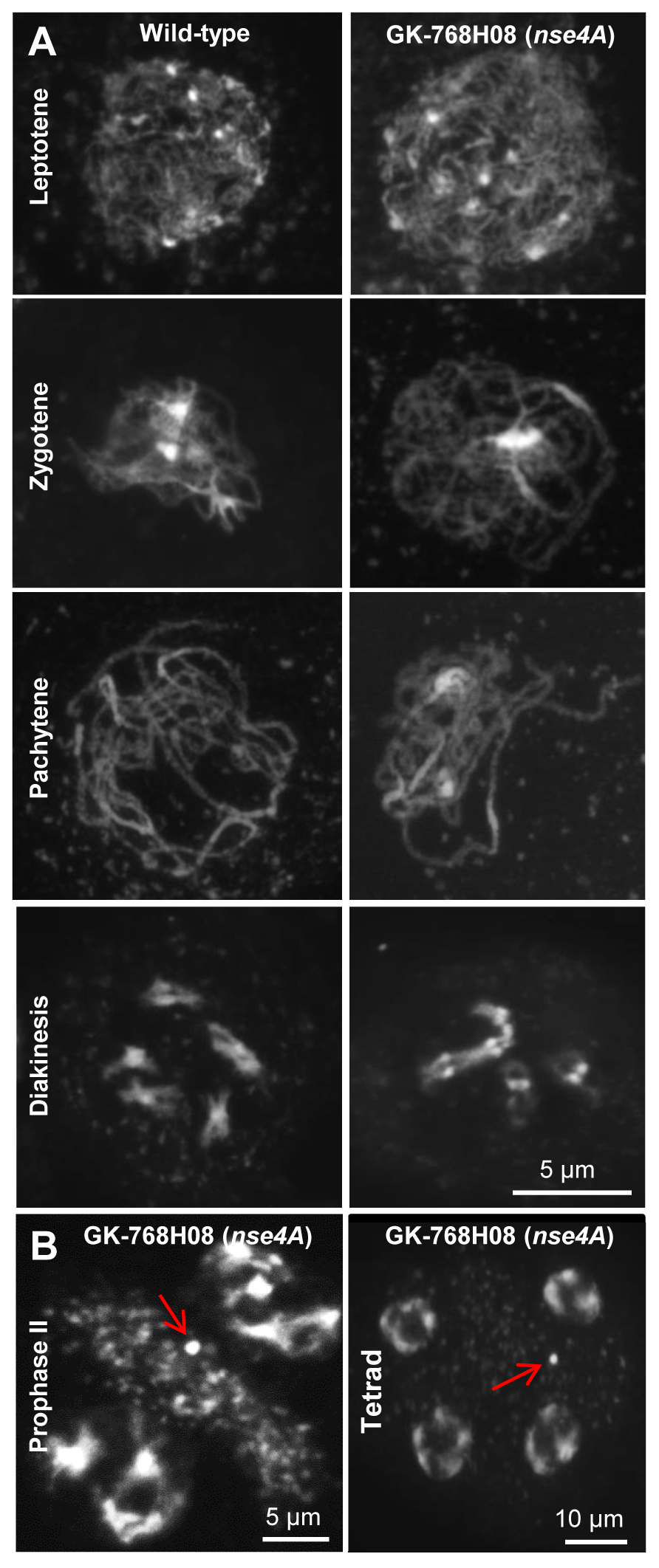

Supplement: FIGURE S1 — Amino acids sequence alignment between full-length NSE4A and NSE4B. The alignment was performed by the Clustal Omega 2.1 software (https://www.ebi.ac.uk/Tools/msa/clustalo/). ∗, Identical amino acids; :, similar amino acids; –, missing amino acids. The conserved functional protein domains were predicted using the Motif Scan (https://myhits.isb-sib.ch/cgi-bin/motif_scan) and Eukaryotic Linear Motif (http://elm.eu.org/) resources. The putative SMC6-binding domain and the conserved C-terminal NSE4_C domain are highlighted in turquoise and yellow, respectively. The amino acids of putative degradation regions and SUMOlisation sites are labeled in red and in green, respectively. The amino acids “VKPE” marked in blue are a SUMOlisation site overlapping with the amino acids “KPGAGVKPE” of a putative degradation site. The region used to produce recombinant anti-NSE4A antibodies is underlined. NSE4A and NSE4B share 67.7% sequence identity. [file Presentation_1.zip › SupplFig12.tif]

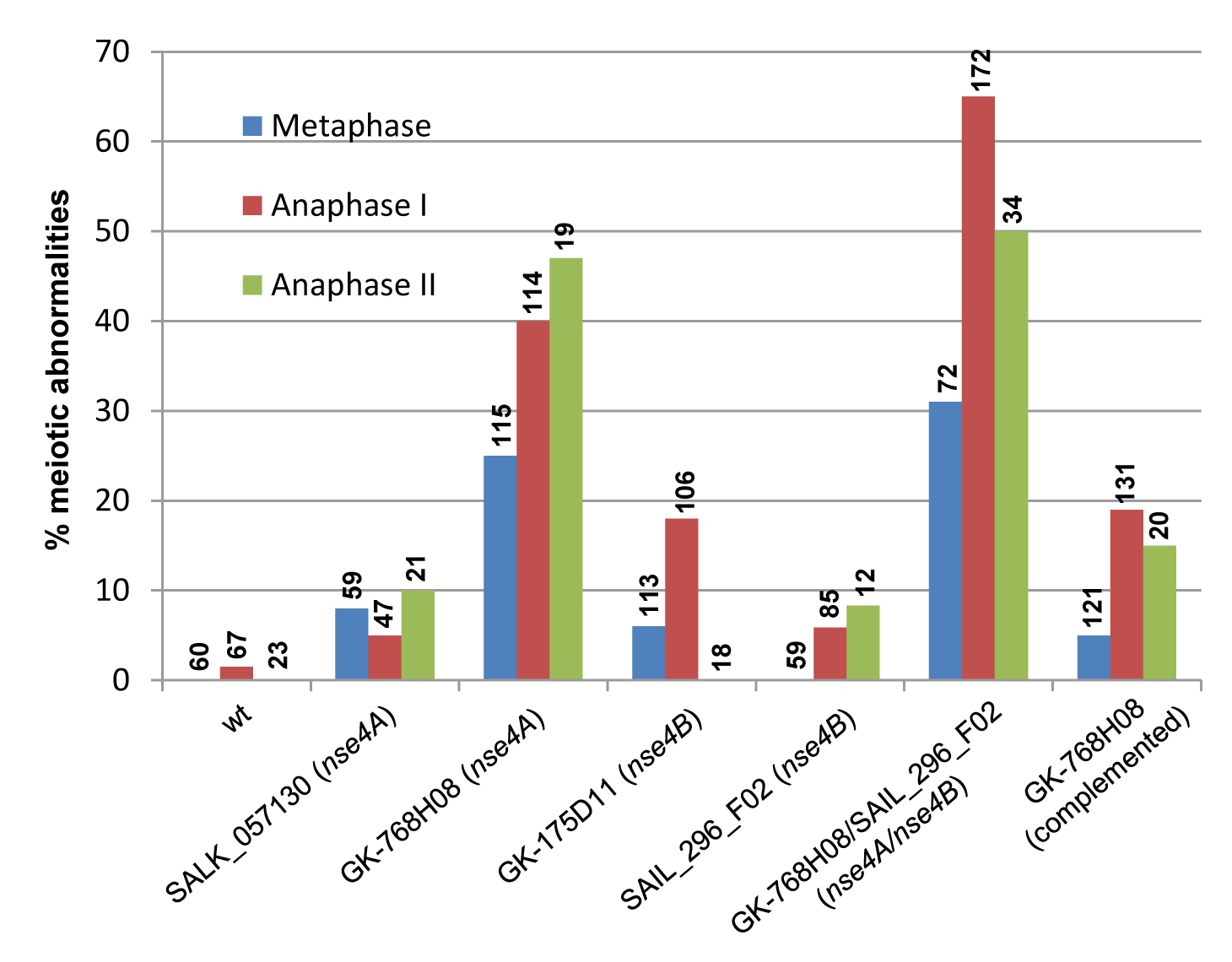

Supplement: FIGURE S1 — Amino acids sequence alignment between full-length NSE4A and NSE4B. The alignment was performed by the Clustal Omega 2.1 software (https://www.ebi.ac.uk/Tools/msa/clustalo/). ∗, Identical amino acids; :, similar amino acids; –, missing amino acids. The conserved functional protein domains were predicted using the Motif Scan (https://myhits.isb-sib.ch/cgi-bin/motif_scan) and Eukaryotic Linear Motif (http://elm.eu.org/) resources. The putative SMC6-binding domain and the conserved C-terminal NSE4_C domain are highlighted in turquoise and yellow, respectively. The amino acids of putative degradation regions and SUMOlisation sites are labeled in red and in green, respectively. The amino acids “VKPE” marked in blue are a SUMOlisation site overlapping with the amino acids “KPGAGVKPE” of a putative degradation site. The region used to produce recombinant anti-NSE4A antibodies is underlined. NSE4A and NSE4B share 67.7% sequence identity. [file Presentation_1.zip › SupplFig13.tif]

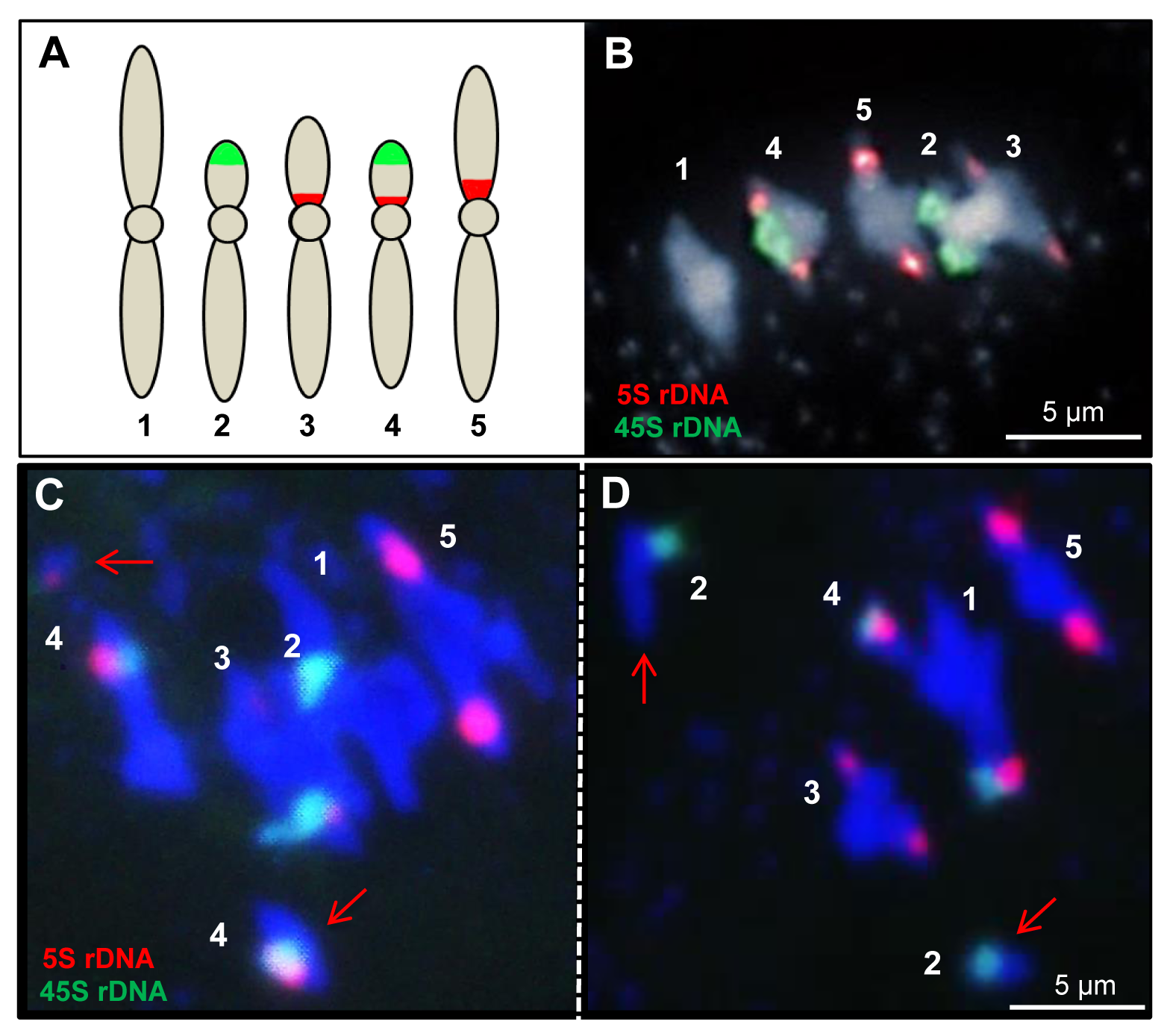

Supplement: FIGURE S1 — Amino acids sequence alignment between full-length NSE4A and NSE4B. The alignment was performed by the Clustal Omega 2.1 software (https://www.ebi.ac.uk/Tools/msa/clustalo/). ∗, Identical amino acids; :, similar amino acids; –, missing amino acids. The conserved functional protein domains were predicted using the Motif Scan (https://myhits.isb-sib.ch/cgi-bin/motif_scan) and Eukaryotic Linear Motif (http://elm.eu.org/) resources. The putative SMC6-binding domain and the conserved C-terminal NSE4_C domain are highlighted in turquoise and yellow, respectively. The amino acids of putative degradation regions and SUMOlisation sites are labeled in red and in green, respectively. The amino acids “VKPE” marked in blue are a SUMOlisation site overlapping with the amino acids “KPGAGVKPE” of a putative degradation site. The region used to produce recombinant anti-NSE4A antibodies is underlined. NSE4A and NSE4B share 67.7% sequence identity. [file Presentation_1.zip › SupplFig14.tif]

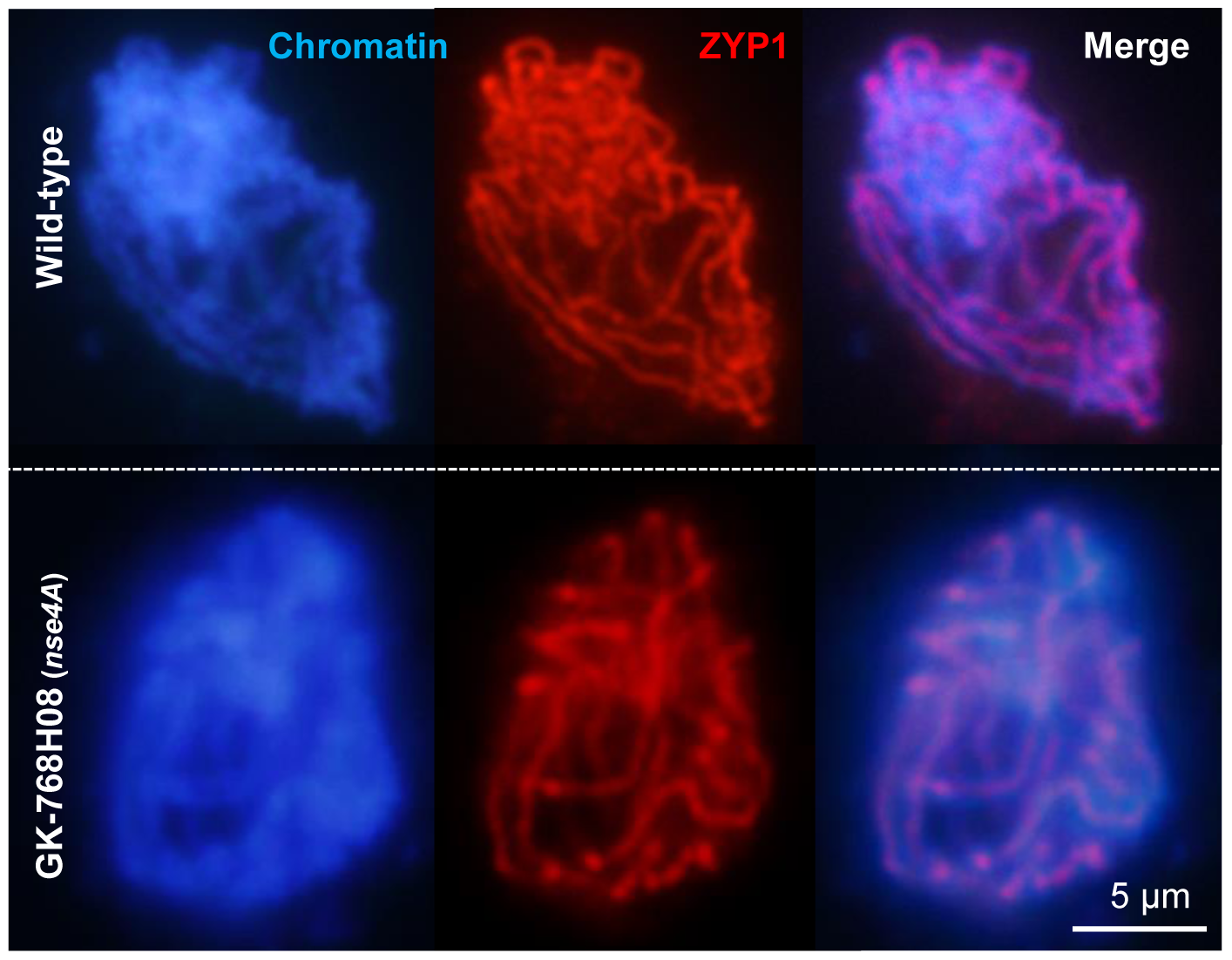

Supplement: FIGURE S1 — Amino acids sequence alignment between full-length NSE4A and NSE4B. The alignment was performed by the Clustal Omega 2.1 software (https://www.ebi.ac.uk/Tools/msa/clustalo/). ∗, Identical amino acids; :, similar amino acids; –, missing amino acids. The conserved functional protein domains were predicted using the Motif Scan (https://myhits.isb-sib.ch/cgi-bin/motif_scan) and Eukaryotic Linear Motif (http://elm.eu.org/) resources. The putative SMC6-binding domain and the conserved C-terminal NSE4_C domain are highlighted in turquoise and yellow, respectively. The amino acids of putative degradation regions and SUMOlisation sites are labeled in red and in green, respectively. The amino acids “VKPE” marked in blue are a SUMOlisation site overlapping with the amino acids “KPGAGVKPE” of a putative degradation site. The region used to produce recombinant anti-NSE4A antibodies is underlined. NSE4A and NSE4B share 67.7% sequence identity. [file Presentation_1.zip › SupplFig15.tif]

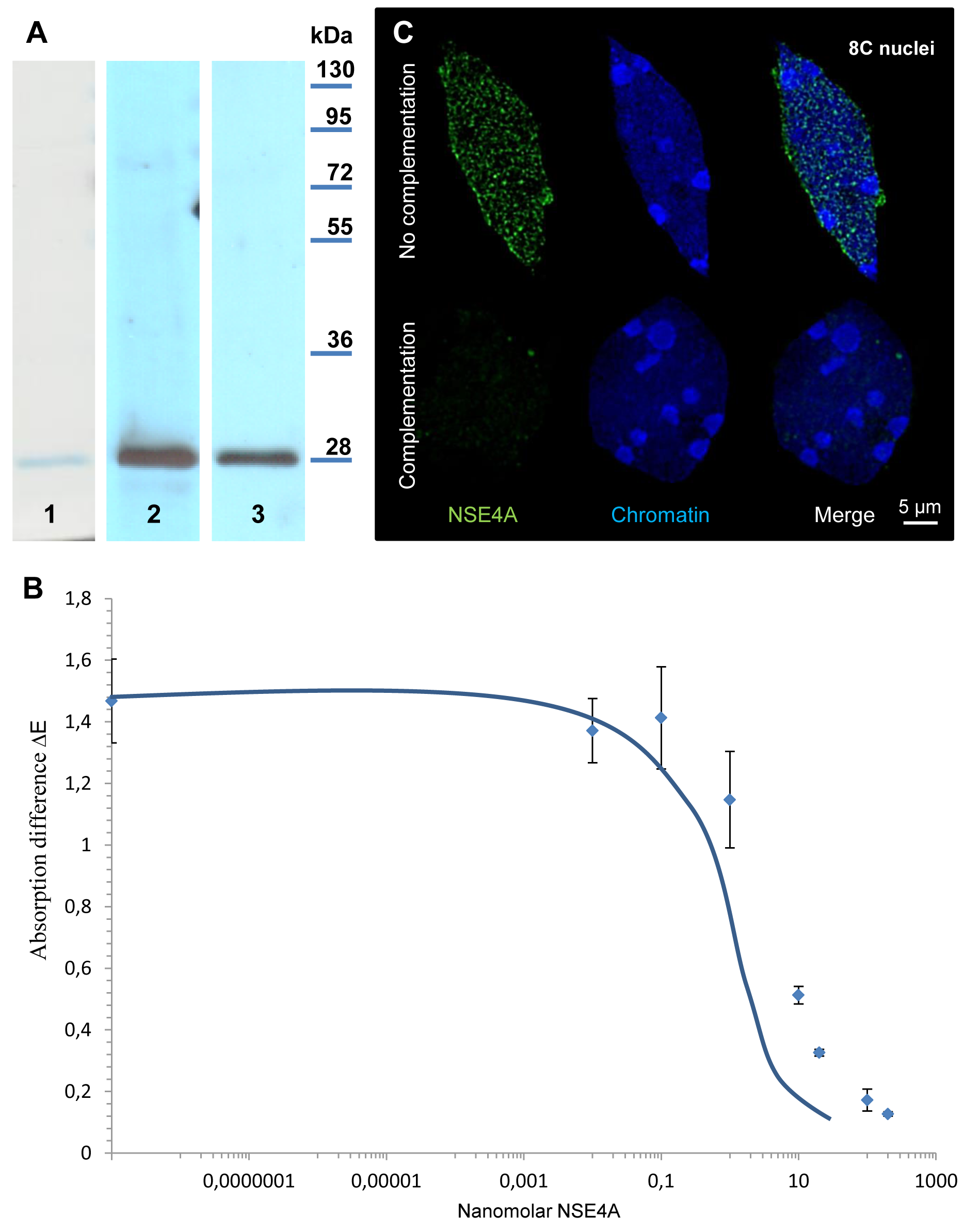

Supplement: FIGURE S1 — Amino acids sequence alignment between full-length NSE4A and NSE4B. The alignment was performed by the Clustal Omega 2.1 software (https://www.ebi.ac.uk/Tools/msa/clustalo/). ∗, Identical amino acids; :, similar amino acids; –, missing amino acids. The conserved functional protein domains were predicted using the Motif Scan (https://myhits.isb-sib.ch/cgi-bin/motif_scan) and Eukaryotic Linear Motif (http://elm.eu.org/) resources. The putative SMC6-binding domain and the conserved C-terminal NSE4_C domain are highlighted in turquoise and yellow, respectively. The amino acids of putative degradation regions and SUMOlisation sites are labeled in red and in green, respectively. The amino acids “VKPE” marked in blue are a SUMOlisation site overlapping with the amino acids “KPGAGVKPE” of a putative degradation site. The region used to produce recombinant anti-NSE4A antibodies is underlined. NSE4A and NSE4B share 67.7% sequence identity. [file Presentation_1.zip › SupplFig2.tif]

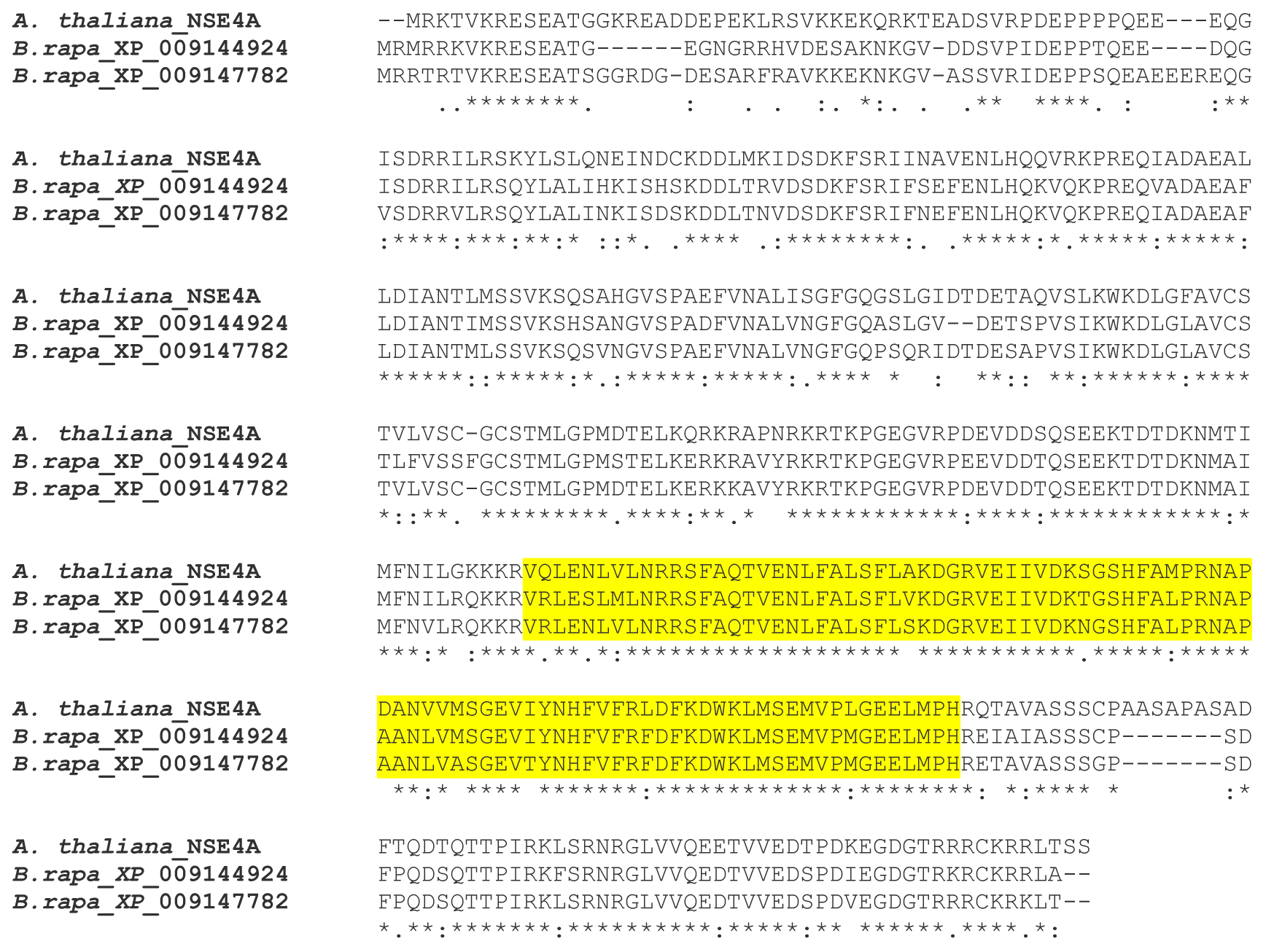

Supplement: FIGURE S1 — Amino acids sequence alignment between full-length NSE4A and NSE4B. The alignment was performed by the Clustal Omega 2.1 software (https://www.ebi.ac.uk/Tools/msa/clustalo/). ∗, Identical amino acids; :, similar amino acids; –, missing amino acids. The conserved functional protein domains were predicted using the Motif Scan (https://myhits.isb-sib.ch/cgi-bin/motif_scan) and Eukaryotic Linear Motif (http://elm.eu.org/) resources. The putative SMC6-binding domain and the conserved C-terminal NSE4_C domain are highlighted in turquoise and yellow, respectively. The amino acids of putative degradation regions and SUMOlisation sites are labeled in red and in green, respectively. The amino acids “VKPE” marked in blue are a SUMOlisation site overlapping with the amino acids “KPGAGVKPE” of a putative degradation site. The region used to produce recombinant anti-NSE4A antibodies is underlined. NSE4A and NSE4B share 67.7% sequence identity. [file Presentation_1.zip › SupplFig3.tif]

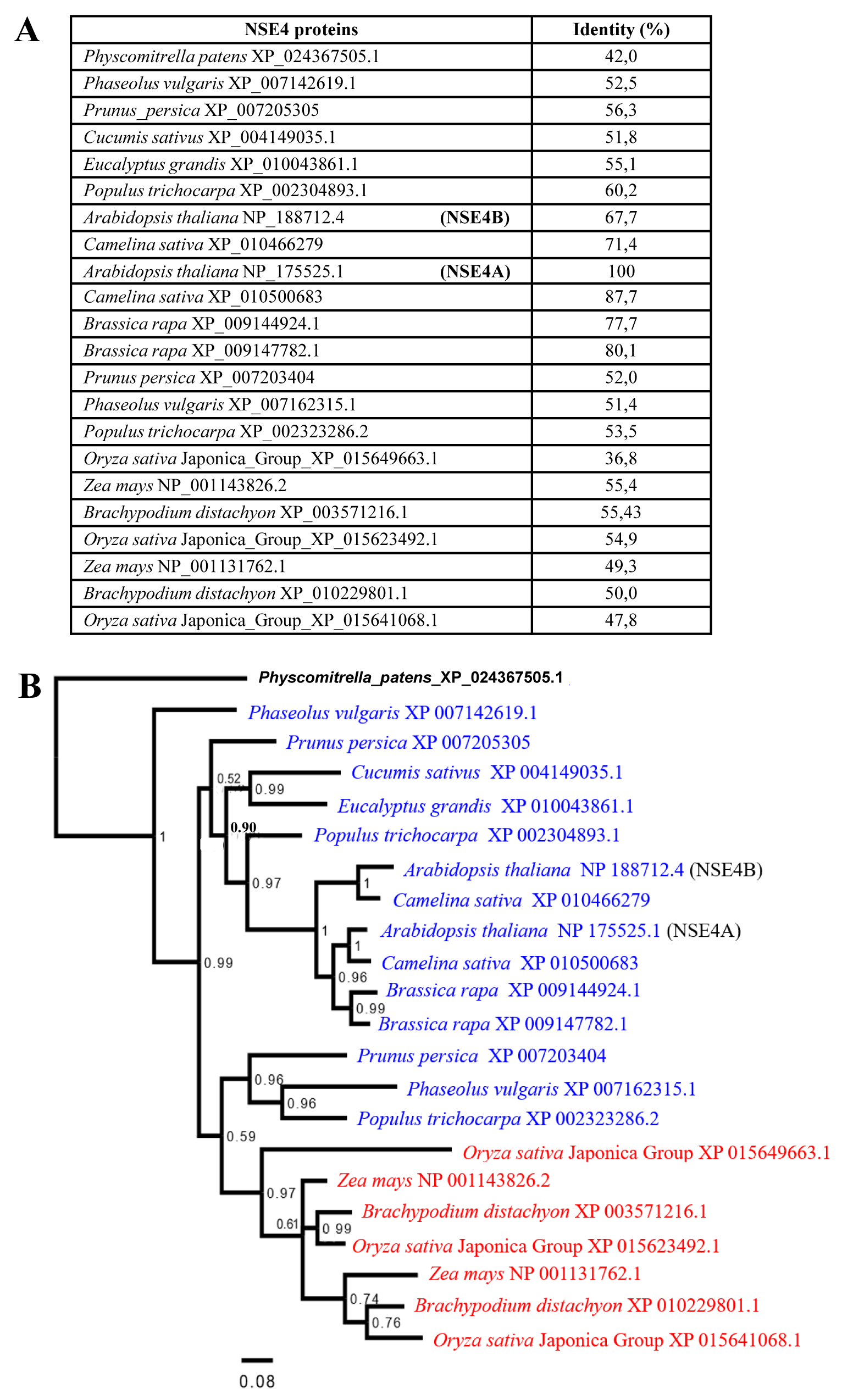

Supplement: FIGURE S1 — Amino acids sequence alignment between full-length NSE4A and NSE4B. The alignment was performed by the Clustal Omega 2.1 software (https://www.ebi.ac.uk/Tools/msa/clustalo/). ∗, Identical amino acids; :, similar amino acids; –, missing amino acids. The conserved functional protein domains were predicted using the Motif Scan (https://myhits.isb-sib.ch/cgi-bin/motif_scan) and Eukaryotic Linear Motif (http://elm.eu.org/) resources. The putative SMC6-binding domain and the conserved C-terminal NSE4_C domain are highlighted in turquoise and yellow, respectively. The amino acids of putative degradation regions and SUMOlisation sites are labeled in red and in green, respectively. The amino acids “VKPE” marked in blue are a SUMOlisation site overlapping with the amino acids “KPGAGVKPE” of a putative degradation site. The region used to produce recombinant anti-NSE4A antibodies is underlined. NSE4A and NSE4B share 67.7% sequence identity. [file Presentation_1.zip › SupplFig4.tif]

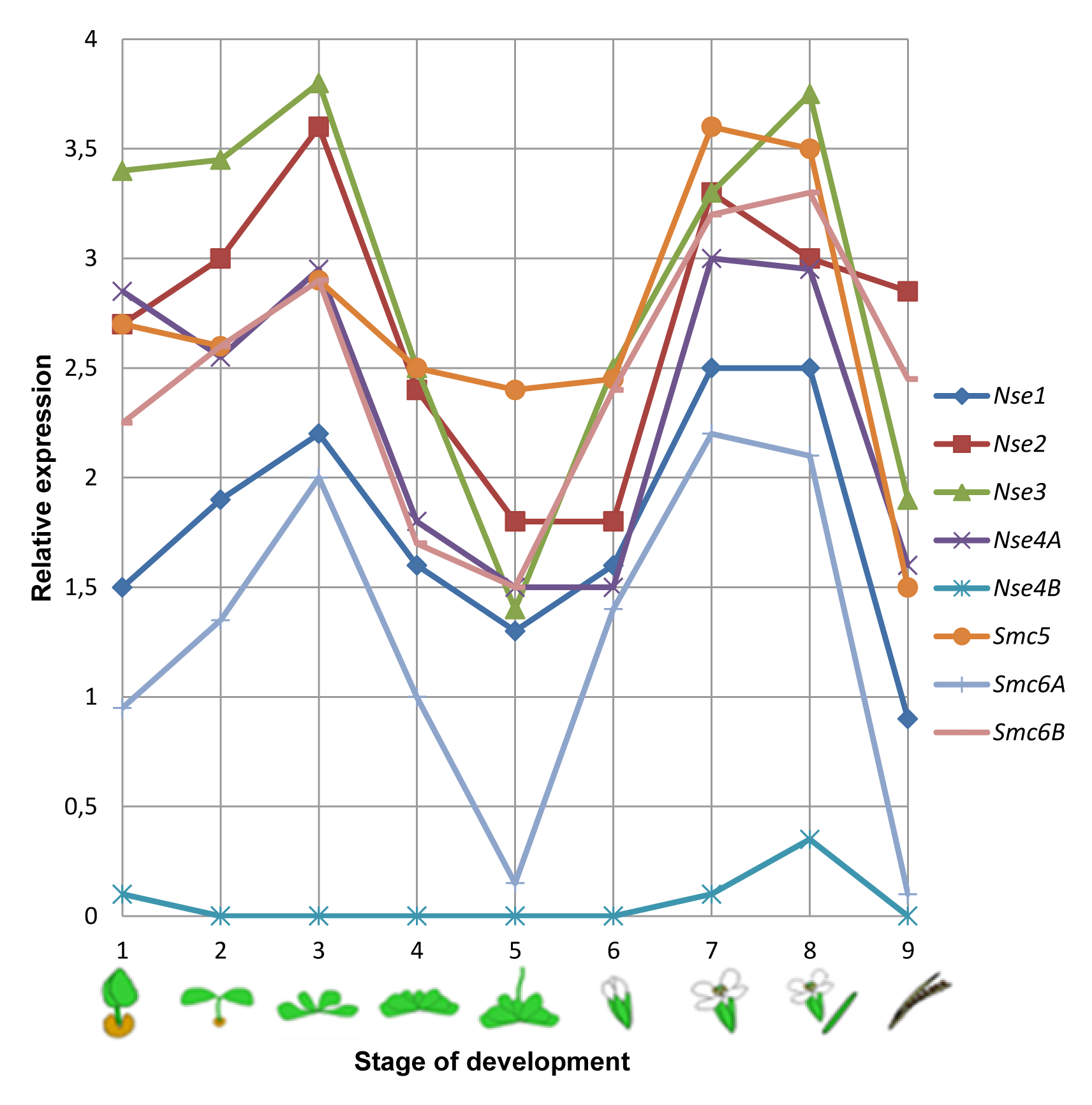

Supplement: FIGURE S1 — Amino acids sequence alignment between full-length NSE4A and NSE4B. The alignment was performed by the Clustal Omega 2.1 software (https://www.ebi.ac.uk/Tools/msa/clustalo/). ∗, Identical amino acids; :, similar amino acids; –, missing amino acids. The conserved functional protein domains were predicted using the Motif Scan (https://myhits.isb-sib.ch/cgi-bin/motif_scan) and Eukaryotic Linear Motif (http://elm.eu.org/) resources. The putative SMC6-binding domain and the conserved C-terminal NSE4_C domain are highlighted in turquoise and yellow, respectively. The amino acids of putative degradation regions and SUMOlisation sites are labeled in red and in green, respectively. The amino acids “VKPE” marked in blue are a SUMOlisation site overlapping with the amino acids “KPGAGVKPE” of a putative degradation site. The region used to produce recombinant anti-NSE4A antibodies is underlined. NSE4A and NSE4B share 67.7% sequence identity. [file Presentation_1.zip › SupplFig5.tif]

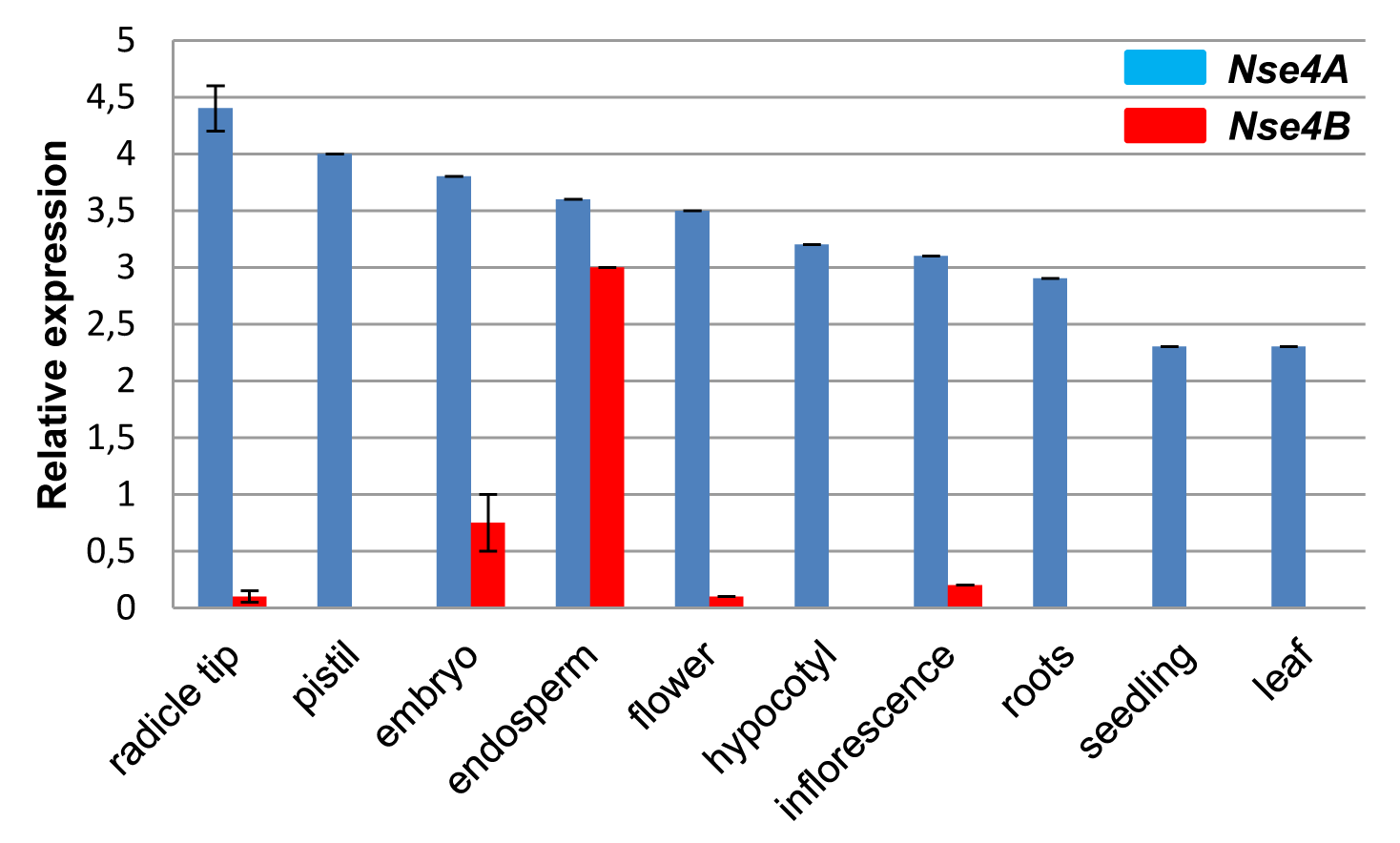

Supplement: FIGURE S1 — Amino acids sequence alignment between full-length NSE4A and NSE4B. The alignment was performed by the Clustal Omega 2.1 software (https://www.ebi.ac.uk/Tools/msa/clustalo/). ∗, Identical amino acids; :, similar amino acids; –, missing amino acids. The conserved functional protein domains were predicted using the Motif Scan (https://myhits.isb-sib.ch/cgi-bin/motif_scan) and Eukaryotic Linear Motif (http://elm.eu.org/) resources. The putative SMC6-binding domain and the conserved C-terminal NSE4_C domain are highlighted in turquoise and yellow, respectively. The amino acids of putative degradation regions and SUMOlisation sites are labeled in red and in green, respectively. The amino acids “VKPE” marked in blue are a SUMOlisation site overlapping with the amino acids “KPGAGVKPE” of a putative degradation site. The region used to produce recombinant anti-NSE4A antibodies is underlined. NSE4A and NSE4B share 67.7% sequence identity. [file Presentation_1.zip › SupplFig6.tif]

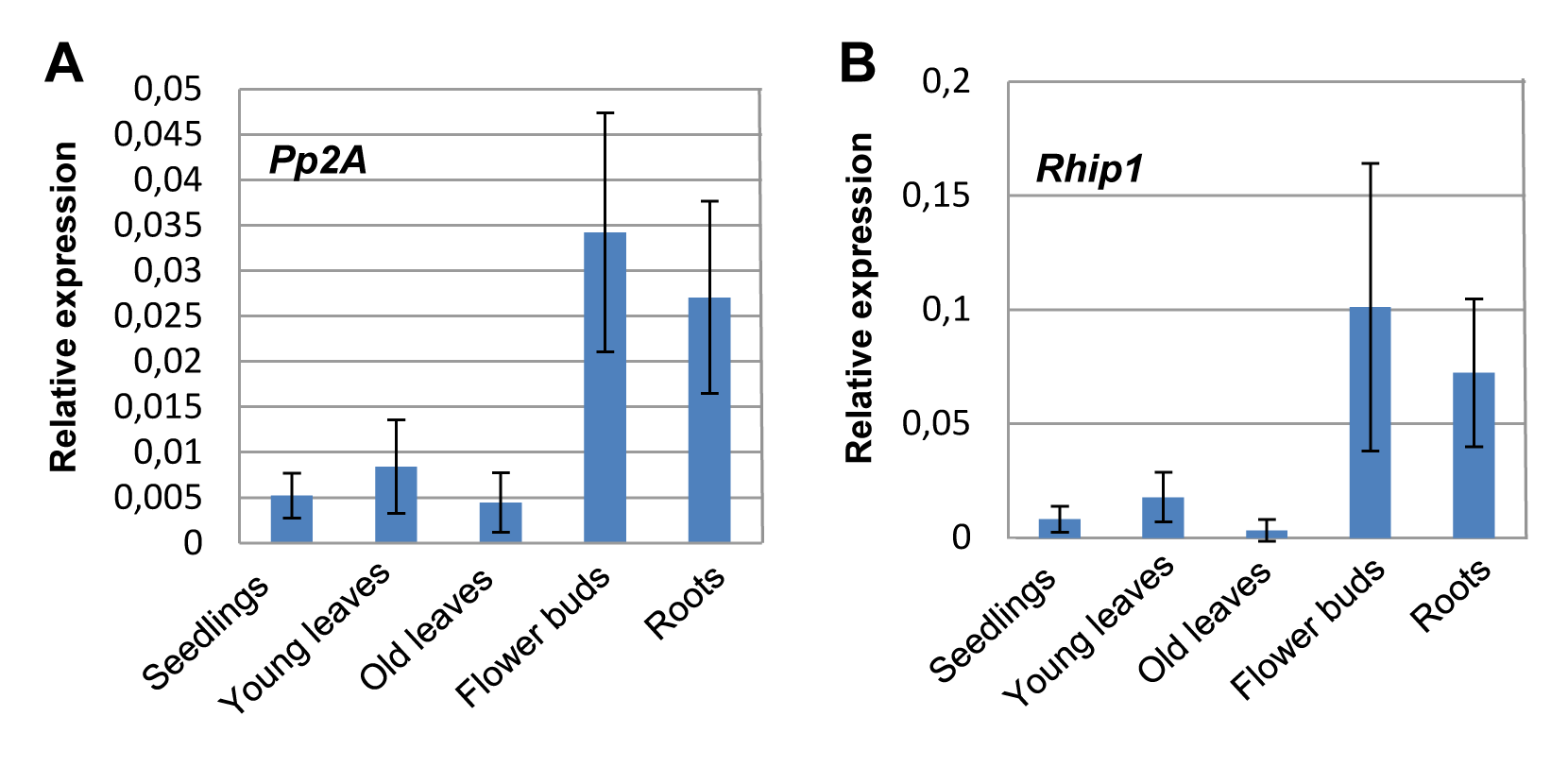

Supplement: FIGURE S1 — Amino acids sequence alignment between full-length NSE4A and NSE4B. The alignment was performed by the Clustal Omega 2.1 software (https://www.ebi.ac.uk/Tools/msa/clustalo/). ∗, Identical amino acids; :, similar amino acids; –, missing amino acids. The conserved functional protein domains were predicted using the Motif Scan (https://myhits.isb-sib.ch/cgi-bin/motif_scan) and Eukaryotic Linear Motif (http://elm.eu.org/) resources. The putative SMC6-binding domain and the conserved C-terminal NSE4_C domain are highlighted in turquoise and yellow, respectively. The amino acids of putative degradation regions and SUMOlisation sites are labeled in red and in green, respectively. The amino acids “VKPE” marked in blue are a SUMOlisation site overlapping with the amino acids “KPGAGVKPE” of a putative degradation site. The region used to produce recombinant anti-NSE4A antibodies is underlined. NSE4A and NSE4B share 67.7% sequence identity. [file Presentation_1.zip › SupplFig7.tif]

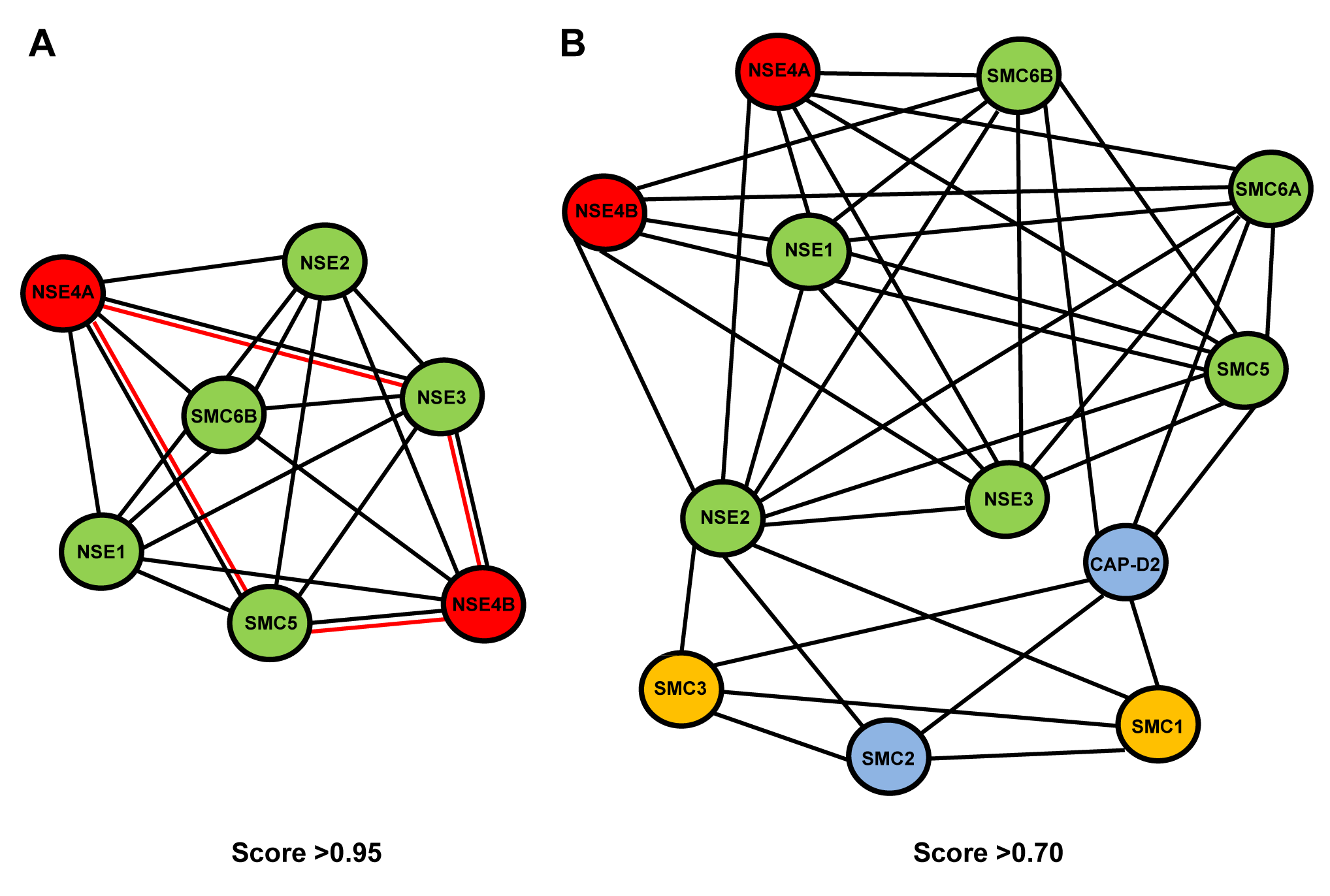

Supplement: FIGURE S1 — Amino acids sequence alignment between full-length NSE4A and NSE4B. The alignment was performed by the Clustal Omega 2.1 software (https://www.ebi.ac.uk/Tools/msa/clustalo/). ∗, Identical amino acids; :, similar amino acids; –, missing amino acids. The conserved functional protein domains were predicted using the Motif Scan (https://myhits.isb-sib.ch/cgi-bin/motif_scan) and Eukaryotic Linear Motif (http://elm.eu.org/) resources. The putative SMC6-binding domain and the conserved C-terminal NSE4_C domain are highlighted in turquoise and yellow, respectively. The amino acids of putative degradation regions and SUMOlisation sites are labeled in red and in green, respectively. The amino acids “VKPE” marked in blue are a SUMOlisation site overlapping with the amino acids “KPGAGVKPE” of a putative degradation site. The region used to produce recombinant anti-NSE4A antibodies is underlined. NSE4A and NSE4B share 67.7% sequence identity. [file Presentation_1.zip › SupplFig8.tif]

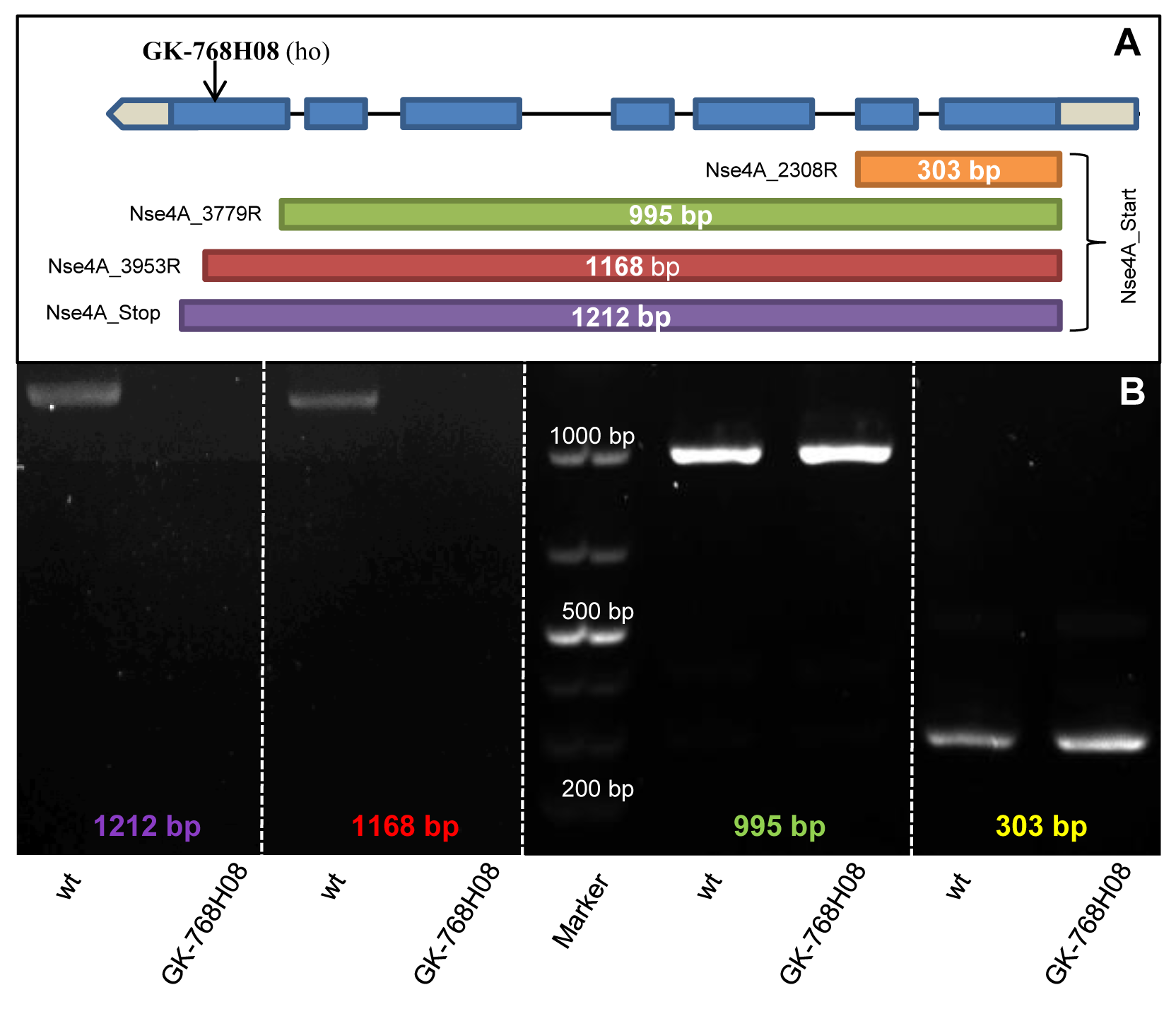

Supplement: FIGURE S1 — Amino acids sequence alignment between full-length NSE4A and NSE4B. The alignment was performed by the Clustal Omega 2.1 software (https://www.ebi.ac.uk/Tools/msa/clustalo/). ∗, Identical amino acids; :, similar amino acids; –, missing amino acids. The conserved functional protein domains were predicted using the Motif Scan (https://myhits.isb-sib.ch/cgi-bin/motif_scan) and Eukaryotic Linear Motif (http://elm.eu.org/) resources. The putative SMC6-binding domain and the conserved C-terminal NSE4_C domain are highlighted in turquoise and yellow, respectively. The amino acids of putative degradation regions and SUMOlisation sites are labeled in red and in green, respectively. The amino acids “VKPE” marked in blue are a SUMOlisation site overlapping with the amino acids “KPGAGVKPE” of a putative degradation site. The region used to produce recombinant anti-NSE4A antibodies is underlined. NSE4A and NSE4B share 67.7% sequence identity. [file Presentation_1.zip › SupplFig9.tif]
