# Supplementary material for: Arabidopsis NSE4 Proteins Act in Somatic Nuclei and Meiosis to Ensure Plant Viability and Fertility
Source: Front Plant Sci. 2019 Jun 20;10:774. doi: 10.3389/fpls.2019.00774 (PMC6596448; doi:10.3389/fpls.2019.00774)
Supplement: TABLE S1 — Primers used to identify the T-DNA insertion alleles. [file Data_Sheet_1.docx]

**Table S1** Primers used to identify the T-DNA insertion alleles.

| Gene | T-DNA line | Primer | Sequence (5`- 3`) |
| --- | --- | --- | --- |
| ***Nse4A*** | GK-768H08 | 1 | GTGGTTCCCGAGGAAGTAAAC |
|  |  | 2 | CAGCTTGAGAATTTGGTGCTC |
|  | Sail-71-A08 | 3 | AAAATCCCCAAATTCAACCTG |
|  |  | 4 | TACCTCATCTGGACGAACACC |
|  | Salk-057130 | 5 | AAACACCACTGATCGATCAGG |
|  |  | 6 | AAAATCCCCAAATTCAACCTG |
| **Nse4B** | GK-175D11 | 7 | TGCTATTCCAGAAATGTTGCC |
|  |  | 8 | CAGAAAACGTACAAAGCCTGG |
|  | Sail-296F02 | 9 | TTTGTACGTTTTCTGTTGCCC |
|  |  | 10 | GTTTCACCTTTTTCCTTTCCG |
|  | T-DNA primers | SALK_LB1.3 | ATTTTGCCGATTTCGGAAC |
|  |  | SAIL_LB3 | GCCTTTTCAGAAATGGATAAATAGCCTTGCTTCC |
|  |  | GK LB | ATAATAACGCTGCGGACATCTACATTTT |

**Table S2** Sequences of the left border junctions of the T-DNA insertion lines. The red letters represent the sequence derived from the T-DNA, and their position in each of the sequences reflects the orientation of the inserted T-DNA.

| Gene | T-DNA line | Sequence (5’ - 3’) |
| --- | --- | --- |
| **AT1G51130 (*Nse4A*)** | GK-768H08 | TGAATATATCCTGATCTCATCAGCGGGACAAGAAGATGA |
|  | SAIL-71-A08 | TTCAGTATCCATAGGTCCCAACCTGGTAACAAAACAAATT |
|  | Salk-057130 | AGAATTATTAATGCAGTTGAAAACAAATTGACGTTTAGACA |
| **AT3G20760 (*Nse4B*)** | SAIL-296-F02 | GCTCTTACCCACAAGATCAAAGGTTACATTCTATTTCTTCTA |
|  | GK-175D11 | TTGGTGCTCAATAGGATATATTCAATTGTAAATGGCTTCAT |

**Table S3** Quantitative real-time RT-PCR primers used to amplify transcripts

| Gene | Primer | Sequences (5`- 3`) |
| --- | --- | --- |
| **AT1G51130 (*Nse4A*)** | I | CTTGCACCAACAAGTTCGGA |
|  | II | GCAGACTGAGACTTCACCGA |
| **AT3G20760 (*Nse4B*)** | III | TGCACCAGAAAGTTCGGAAG |
|  | IV | AGCTGATTGCGATTTCACCG |
| **AT1G13320** (Pp2A reference gene) |  | TAACGTGGCCAAAATGATGC |
|  |  | GTTCTCCACAACCGCTTGGT |
| **AT4G26410** (Rhip1 reference gene) |  | GAGCTGAAGTGGCTTCCATGAC |
|  |  | GGTCCGACATACCCATGATCC |

**Table S4** Primers used to clone the *Nse4A* genes, to produce clones for recombinant protein expression, and for the transcript analyses of the mutants and transgenic lines

|  | **Primer** | **PCR product size** | **Sequences (5` - 3`)** |
| --- | --- | --- | --- |
| Cloning | Prom-Nse4A |  | CAATTTCATAAATCTTGAACATATGAC |
|  | Nse4A_Start-Clo |  | TGGATCCTTATGAGGAAGACGGTGAAGC |
|  | Nse4A_Stop-Clo |  | AACTCGAGGCTTAAGAAGAGGTGAGTCTCCGC |
|  | Nse4A_StopMiss |  | AACTCGAGGCAGAGGTGAGTCTCCGCTTACAT |
| Recombinant protein | Nse4a_AB_For |  | TGGATCCCCTCAGGAGGAAGAACAAGG |
|  | Nse4a_AB_rev |  | AACTCGAGCATGGCGAAATGAGAACCAC |
| Transcript analyses of  GK-768H08 | GFP (Start) |  | ATGGTGAGCAAGGGCGAG |
|  | GFP (Stop) |  | TTACTTGTACAGCTCGTCCATG |
|  | Nse4A_2308R |  | TTGTTGGTGCAAGTTTTCAA |
|  | Nse4A_3779R |  | ATTAGCTCCTCGCCCAGTG |
|  | Nse4A_3953R |  | CGCCCTCTTTGTCAGGAGTA |

**Table S5** *A. thaliana* NSE4 protein sequence identities (%) compared to orthologs of non-plant organisms. The matrix was generated by the Clustal Omega 2.1 software.

|  | *A t* (NSE4A) | *At* (NSE4B)B) | *Sc* | *Eh* | *Dd* | *Mm*  (NSE4A) ) | *Hs*  (NSE4A) | *Mm*  (NSE4B) | *Hs*  (NSE4B) |
| --- | --- | --- | --- | --- | --- | --- | --- | --- | --- |
| *At* (NSE4A) | 100 | 67.7 | 17.6 | 21.6 | 23.5 | 21.5 | 20.1 | 21.0 | 18.2 |
| *At* (NSE4B) |  | 100 | 16.7 | 22.1 | 20.6 | 20.7 | 21.4 | 20.1 | 18.1 |
| *Sc* |  | | 100 | 18.3 | 18.7 | 23.6 | 23.6 | 21.2 | 21.7 |
| *Eh* |  | | | 100 | 21.2 | 21.7 | 21.7 | 24.0 | 18.7 |
| *Dd* |  | | | | 100 | 25.3 | 24.7 | 22.8 | 19.5 |
| *Mm* (NSE4A) |  | | | | | 100 | 88.2 | 48.6 | 43.6 |
| *Hs* (NSE4A) |  | | | | | | 100 | 47.8 | 43.1 |
| *Mm* (NSE4B) |  | | | | | | | 100 | 64.6 |
| *Hs* (NSE4B) |  | | | | | | |  | 100 |

*At - A. thaliana*, *Sc*– *S. cerevisiae*, *Eh* – *Entamoeba histolytica*, Dd – *Dictyostelium discoideum*; *Mm* – *M. musculus*, Hs – *H. sapiens*

**Table S6** Genes showing high co-expression with *Nse4A* predicted from18 different anatomical tissues. The data were obtained from the AT_mRNASeq_ARABI_GL-0 database of https://genevestigator.com. Scores indicate the level of correlation of expression in different anatomical samples. Bold gene names indicate meiosis- or chromatin-related genes.

| **Score** | **Gene name** | **Description** |
| --- | --- | --- |
| 0.96 | MED8 | Mediator of RNA polymerase II transcription subunit 8 |
| 0.96 | AT3G27120 |  |
| 0.96 | AT1G07970 |  |
| 0.95 | AT5G10900 |  |
| 0.95 | SMU1 | Suppressor of mec-8 and unc-52 protein homolog 1 |
| 0.95 | AT2G04235 |  |
| 0.95 | TRO | Protein TRAUCO |
| 0.95 | **DRT111** | DNA-damage-repair/toleration protein |
| 0.95 | AT4G39600 |  |
| 0.95 | **APC5** | Anaphase-promoting complex subunit 5 |
| 0.94 | AT3G20010 |  |
| 0.94 | **CHR17** | Chromatin remodeling factor17 |
| 0.94 | AT3G07300 |  |
| 0.94 | AT3G19120 |  |
| 0.94 | **SUVH4** | Histone-lysine N-methyltransferase |
| 0.94 | THO6 | THO complex subunit 6 |
| 0.94 | **HDA15** | Histone deacetylase 15 |
| 0.94 | AT3G27420 |  |
| 0.94 | AT2G06040 |  |
| 0.94 | **RFC3** | Replication factor C subunit 3 |
| 0.94 | AT5G40400 |  |
| 0.94 | **MLH1** | DNA mismatch repair protein MLH1 |
| 0.94 | PCMP-E32 | Putative pentatricopeptide repeat containing protein |
| 0.94 | AT2G28330 |  |
| 0.94 | AT4G14490 |  |
| 0.94 | NUP93B | Nuclear pore complex protein NUP93B |
| 0.94 | AT2G43190 |  |
| 0.93 | NUP155 | Nuclear pore complex protein NUP155 |
| 0.93 | ATR | Serine/threonine-protein kinase ATR |
| 0.93 | **MSH3** | DNA mismatch repair protein MSH3 |
| 0.93 | **ATX1** | Histone-lysine N-methyltransferase ATX1 |
| 0.93 | AT5G15570 |  |
| 0.93 | TIFY4A | Protein TIFY 4A |
| 0.93 | FIO1 | methyltransferases |
| 0.93 | SNRNP25 | U11/U12 small nuclear ribonucleoprotein 25kDa |
| 0.93 | HAL3A | Phosphopantothenoylcysteine decarboxylase |
| 0.93 | AT4G24320 |  |
| 0.93 | AT5G45720 |  |
| 0.93 | POLD1 | DNA polymerase delta catalytic subunit |
| 0.93 | CYCA3-4 | Cyclin-A3-4 |
| 0.93 | AT5G49110 |  |
| 0.93 | PCMP-E76 | Pentatricopeptide repeat-containing protein |
| 0.93 | AT4G00525 |  |
| 0.93 | AT3G26750 |  |
| 0.93 | AT1G66345 |  |
| 0.93 | AUR3 | Serine/threonine-protein kinase Aurora-3 |
| 0.93 | AT3G24860 |  |
| 0.93 | **APC7** | Anaphase-promoting complex subunit 7 |
| 0.93 | AT3G58930 |  |
| 0.93 | AT5G47660 |  |
| 0.93 | AT1G64583 |  |
| 0.92 | GLE1 | Protein GLE1 |
| 0.92 | **APC4** | Anaphase-promoting complex subunit 4 |
| 0.92 | SPT | Transcription factor SPATULA |
| 0.92 | SHH2 | Protein SWADEE HOMEODOMAIN homolog 2 |
| 0.92 | TRFL5 | TRF-like 5 |
| 0.92 | MRLK | Meristematic receptor-like kinase |
| 0.92 | **APC1** | Anaphase-promoting complex subunit 1 |
| 0.92 | ORC4 | Origin recognition complex subunit 4 |
| 0.92 | RAD5 | Putative SWI/SNF protein |
| 0.91 | **SMC2** | Condensin subunit |
